# IRG1 and Inducible Nitric Oxide Synthase Act Redundantly with Other Interferon-Gamma-Induced Factors To Restrict Intracellular Replication of Legionella pneumophila

**DOI:** 10.1128/mBio.02629-19

**Published:** 2019-11-12

**Authors:** Jordan V. Price, Daniel Russo, Daisy X. Ji, Roberto A. Chavez, Lucian DiPeso, Angus Yiu-Fai Lee, Jörn Coers, Russell E. Vance

**Affiliations:** a Department of Biology, Oberlin College, Oberlin, Ohio, USA; b Division of Immunology and Pathogenesis, Department of Molecular and Cell Biology, University of California, Berkeley, California, USA; c Cancer Research Laboratory, University of California, Berkeley, California, USA; d Department of Molecular Genetics and Microbiology, Duke University Medical Center, Durham, North Carolina, USA; e Howard Hughes Medical Institute, University of California, Berkeley, California, USA; Sequella, Inc.

**Keywords:** *Legionella pneumophila*, host-pathogen interactions, innate immunity, interferons, macrophages

## Abstract

Interferon gamma (IFN-γ) restricts the intracellular replication of many pathogens, but the mechanism by which IFN-γ confers cell-intrinsic pathogen resistance remains unclear. For example, intracellular replication of the bacterial pathogen Legionella pneumophila in macrophages is potently curtailed by IFN-γ. However, consistent with prior studies, no individual genetic deficiency that we tested completely abolished IFN-γ-mediated control. Intriguingly, we observed that the glycolysis inhibitor 2-deoxyglucose (2DG) partially rescued L. pneumophila replication in IFN-γ-treated macrophages. 2DG inhibits glycolysis and triggers the unfolded protein response, but unexpectedly, it appears these effects are not responsible for perturbing the antimicrobial activity of IFN-γ. Instead, we found that 2DG rescues bacterial replication by inhibiting the expression of two key antimicrobial factors, inducible nitric oxide synthase (iNOS) and immune-responsive gene 1 (IRG1). Using immortalized and primary macrophages deficient in iNOS and IRG1, we confirmed that loss of both iNOS and IRG1, but not individual deficiency in either gene, partially reduced IFN-γ-mediated restriction of L. pneumophila. Further, using a combinatorial CRISPR/Cas9 mutagenesis approach, we found that mutation of iNOS and IRG1 in combination with four other genes (CASP11, IRGM1, IRGM3, and NOX2) resulted in a total loss of L. pneumophila restriction by IFN-γ in primary bone marrow macrophages. Our study defines a complete set of cell-intrinsic factors required for IFN-γ-mediated restriction of an intracellular bacterial pathogen and highlights the combinatorial strategy used by hosts to block bacterial replication in macrophages.

## Abstract (precis)

Legionella pneumophila is one example among many species of pathogenic bacteria that replicate within mammalian macrophages during infection. The immune signaling factor interferon gamma (IFN-γ) blocks L. pneumophila replication in macrophages and is an essential component of the immune response to L. pneumophila and other intracellular pathogens. However, to date, no study has identified the exact molecular factors induced by IFN-γ that are required for its activity. We generated macrophages lacking different combinations of IFN-γ-induced genes in an attempt to find a genetic background in which there is a complete loss of IFN-γ-mediated restriction of L. pneumophila. We identified six genes that comprise the totality of the IFN-γ-dependent restriction of L. pneumophila replication in macrophages. Our results clarify the molecular basis underlying the potent effects of IFN-γ and highlight how redundancy downstream of IFN-γ is key to prevent exploitation of macrophages by pathogens.

## INTRODUCTION

Macrophages are preferred host cells for many species of intracellular bacterial pathogen. Bona fide pathogens of mammals, such as Mycobacterium tuberculosis, Listeria monocytogenes, and Salmonella enterica, as well as environmental microorganisms that are “accidental” pathogens of mammals, such as Legionella pneumophila, display the ability to replicate efficiently in macrophages, demonstrating that these cells can provide a plastic niche suitable to the metabolic needs of distinct bacterial species (1). To defend against potential exploitation by diverse pathogens, including environmental microorganisms with which they have not coevolved, macrophages require potent mechanisms to restrict intracellular bacterial replication. A cornerstone of the immune response to many intracellular pathogens is the cytokine interferon gamma (IFN-γ). The importance of IFN-γ is highlighted by the observation that genetic deficiencies in the IFN-γ signaling pathway render humans highly susceptible to infections by intracellular pathogens, most notably M. tuberculosis and even normally benign environmental bacteria (2). Mice with deficiencies in the IFN-γ pathway are also highly susceptible to intracellular bacterial pathogens, including M. tuberculosis, L. monocytogenes, S. enterica, Brucella abortus, and L. pneumophila, among others (3–9).

L. pneumophila normally replicates in protozoan host amoebae but can cause a severe pneumonia in humans, known as Legionnaires’ disease, through infection of lung macrophages. L. pneumophila employs a type IV secretion system to translocate bacterial effector proteins into the host cytosol, allowing the bacteria to establish an intracellular replicative compartment (10). Flagellin produced by wild-type L. pneumophila can trigger host cell pyroptosis via the NAIP/NLRC4 inflammasome; however, L. pneumophila bacteria that lack flagellin (Δ*flaA*) are able to replicate to high levels in macrophages (11–17). Brown et al. demonstrated that failure of IFN-γ-deficient mice to control L. pneumophila likely occurs at the level of cell-intrinsic restriction of bacteria in monocyte-derived macrophages that infiltrate the lung following infection (18). Accordingly, *in vitro* infection models using bone marrow-derived macrophages (BMMs) have enabled meaningful study of the cell-intrinsic immune response to L. pneumophila coordinated by IFN-γ. However, despite several decades of evidence supporting an essential role for IFN-γ in the antimicrobial immune response, the precise mechanisms by which IFN-γ acts to mediate cell-intrinsic control of L. pneumophila and other pathogens remain obscure.

Inducible nitric oxide synthase (iNOS, encoded by the gene *Nos2* in mice) plays a key role in the IFN-γ-dependent response to M. tuberculosis and several other pathogens (19–21). iNOS facilitates the production of nitric oxide (NO), a toxic metabolite with direct antimicrobial activity. NO also acts as a regulator of host responses and coordinates metabolic changes in IFN-γ-stimulated macrophages (22–24). While *Nos2*^−/−^ mice display increased susceptibility to infection by M. tuberculosis, evidence suggests this is not simply due to direct cell-intrinsic antimicrobial effects of NO (25). In addition, the activity of iNOS is not absolutely required to control infection by many pathogens, suggesting that there are redundant iNOS-independent mechanisms that underlie the potency of IFN-γ (26). Strikingly, while L. pneumophila does not display resistance to the effects of NO when cultured in broth, *Nos2*^−/−^ macrophages are not impaired in IFN-γ-dependent restriction of L. pneumophila (27–29). This indicates either that L. pneumophila is resistant to the effects of iNOS/NO during infection or, more likely, that there are redundant factors induced by IFN-γ that can restrict L. pneumophila in the absence of iNOS.

Previous work has attempted to address the possibility of redundancy in the IFN-γ-dependent immune response to L. pneumophila. Pilla et al. generated quadruple knockout (QKO) mice deficient in *Nos2*, *Cybb* (cytochrome *b*_558_ subunit beta, encoding NADPH oxidase 2, also known as NOX2), *Irgm1* (immunity-related GTPase family M member 1), and *Irgm3* (immunity-related GTPase family M member 3), all induced by IFN-γ (28). NOX2 partners with phagosomal oxidase components to generate reactive oxygen species, which, like NO, can cause direct toxicity to phagocytized pathogens in neutrophils and macrophages (30, 31). IRGM1 and IRGM3 are antimicrobial GTPases that participate in the disruption of membrane-bound, pathogen-containing compartments within phagocytes in the case of *Toxoplasma* (32, 33) and *Chlamydia* (34). To date, however, a nonredundant role for IRGM1 and IRGM3 in disruption of the L. pneumophila-containing vacuole has not been established. Remarkably, Pilla et al. observed that macrophages derived from QKO mice retained restriction of L. pneumophila replication when stimulated with IFN-γ (28). This study implicated the bacterial lipopolysaccharide (LPS) detector caspase 11 (CASP11), encoded by the gene *Casp4*, in some of the residual IFN-γ-dependent restriction of L. pneumophila replication in macrophages (28). Upon binding of bacterial lipopolysaccharide in the cytoplasm, CASP11 can trigger host macrophage pyroptosis, an inflammatory form of cell death (35, 36).

Recently, Naujoks et al. implicated immune-responsive gene 1 (IRG1), encoded by the gene *Acod1*, in the IFN-γ-dependent immune response to L. pneumophila, demonstrating that driving *Acod1* expression in macrophages was sufficient to suppress L. pneumophila replication (29). However, this study did not address whether macrophages deficient in IRG1 were impaired in the ability to restrict L. pneumophila when stimulated with IFN-γ. Like iNOS, IRG1 generates a potentially toxic metabolite (itaconate) and contributes to metabolic changes that occur in inflamed macrophages (37–39).

We recently described a mutant strain of L. pneumophila (Δ*flaA* Δ*uhpC*) that is able to replicate in macrophages treated with 2-deoxyglucose (2DG), an inhibitor of mammalian glycolysis (40). This strain allows us to probe the role that host cell metabolism plays in the immune response to L. pneumophila. In the present study, we use a combination of preexisting knockout mouse models, pharmacological treatment with 2DG and other drugs, CRISPR/Cas9 genetic manipulation of immortalized mouse macrophages, and primary BMMs from novel strains of CRISPR/Cas9-engineered mice to survey the factors required for IFN-γ-dependent restriction of L. pneumophila in macrophages. Ultimately, we demonstrate that iNOS and IRG1 are redundant in terms of IFN-γ-dependent restriction of L. pneumophila. Further, we identify six IFN-γ-inducible factors, iNOS, IRG1, CASP11, NOX2, IRGM1, and IRGM3, which are responsible for the entirety of the IFN-γ-dependent restriction of L. pneumophila in macrophages.

## RESULTS

### IFN-γ restricts L. pneumophila replication across a spectrum of immune gene-deficient macrophages.

**FIG 1:**
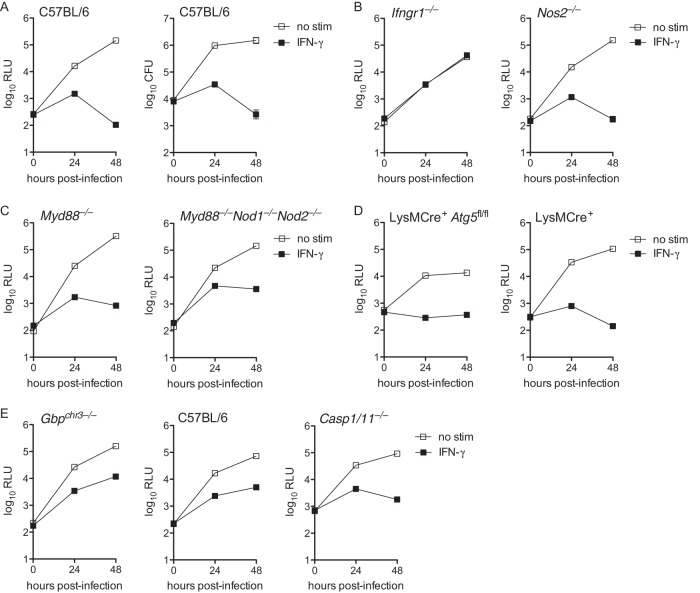
IFN-γ restricts L. pneumophila replication in many different immune gene-deficient macrophages. (A) Luminescence measured in relative light units (log_10_ RLU, left) and recovery of CFU (log_10_ CFU, right) of LP02 Δ*flaA lux* L. pneumophila from infected wild-type C57BL/6 BMMs either not stimulated (no stim) or stimulated with 6.0 ng/ml IFN-γ. (B) Log_10_ RLU from LP02 Δ*flaA lux* L. pneumophila from infected *Ifngr1*^−/−^ and *Nos2*^−/−^ BMMs either not stimulated or stimulated with 6.0 ng/ml IFN-γ. (C) Log_10_ RLU from LP02 Δ*flaA lux* L. pneumophila from infected *Myd88*^−/−^ and *Myd88*^−/−^ *Nod1*^−/−^ *Nod2*^−/−^ BMMs either not stimulated or stimulated with 6.0 ng/ml IFN-γ. (D) Log_10_ RLU from LP02 Δ*flaA lux* L. pneumophila from infected LysMCre^+^ *Atg5*^fl/fl^ and LysMCre^+^ BMMs either not stimulated or stimulated with 6.0 ng/ml IFN-γ. (E) Log_10_ RLU from LP02 Δ*flaA lux* L. pneumophila from infected *Gbp*^chr3−/−^ BMMs, wild-type C57BL/6 control BMMs derived in parallel with *Gbp*^chr3−/−^ BMMs, and *Casp1/11*^−/−^ BMMs either not stimulated or stimulated with 6.0 ng/ml IFN-γ. Data reflect individual experiments that represent at least two independent experiments. Error bars in all graphs represent standard deviation of the mean from at least three technical replicates. *P* < 0.001, comparing no stim versus IFN-γ curves in all genotypes of BMMs (except *Ifngr1*^−/−^) by 2-way analysis of variance (ANOVA). No-stim and IFN-γ curves do not differ significantly in *Ifngr1*^−/−^ BMMs.

In an attempt to identify specific genetic factors that explain the ability of IFN-γ to restrict L. pneumophila replication in macrophages, we tested the ability of IFN-γ to restrict L. pneumophila in BMMs derived from various knockout mice. Using an extensively validated strain of Δ*flaA* L. pneumophila that expresses luminescence (*lux*) genes from Photorhabdus luminescens (28, 40–42), we confirmed that Δ*flaA* L. pneumophila replicates in unstimulated BMMs but does not replicate in BMMs stimulated with IFN-γ (Fig. 1A). As expected, BMMs lacking the IFN-γ receptor (*Ifngr1*^−/−^) did not restrict L. pneumophila replication in the presence of IFN-γ (Fig. 1B). We confirmed that BMMs lacking functional iNOS (*Nos2*^−/−^) retained IFN-γ-dependent restriction of L. pneumophila (Fig. 1B) (27–29). BMMs lacking MYD88 (*Myd88*^−/−^), a key adaptor in the innate inflammatory immune response triggered by bacterial pattern recognition, and BMMs lacking MYD88, NOD1, and NOD2 (*Myd88*^−/−^ *Nod1*^−/−^ *Nod2*^−/−^), which do not activate inflammatory NF-κB signaling in response to L. pneumophila (43), still largely restricted bacterial replication when stimulated with IFN-γ (Fig. 1C). Consistent with previous results (28), BMMs deficient in ATG5 (LysMCre^+^ *Atg5*^fl/fl^), a factor essential for autophagy (44), also mediated IFN-γ-dependent restriction of L. pneumophila replication (Fig. 1D). Pilla et al. reported that guanylate binding proteins (GBPs) in conjunction with CASP11 partially mediated IFN-γ-mediated restriction of L. pneumophila in BMMs (28). We observed similar partial restriction in BMMs from mice that lack a region of chromosome 3 containing five GBPs (GBP1, -2, -3, -5, and -7, *Gbp*^chr3−/−^) and also in BMMs that that lack functional caspase-1 and CASP11 (*Casp1/11*^−/−^) (Fig. 1E). Additionally, we observed that BMMs derived from mice lacking functional MYD88 and TRIF (*Myd88*^−/−^ *Trif*^−/−^), STING (*Goldenticket*) (45), IFNAR (*Ifnar1*^−/−^), and tumor necrosis factor (TNF) receptor (*Tnfr1*^−/−^) all largely retained IFN-γ-dependent restriction of L. pneumophila replication (data not shown). These data do not rule out the possibility that any single genetic factor surveyed here might contribute in part to the restriction of L. pneumophila in IFN-γ-stimulated macrophages. However, our results confirm that no single genetic factor fully accounts for the profound restriction of L. pneumophila observed in IFN-γ-stimulated macrophages.

### 2-Deoxyglucose partially reverses IFN-γ-dependent restriction of L. pneumophila in BMMs.

**FIG 2:**
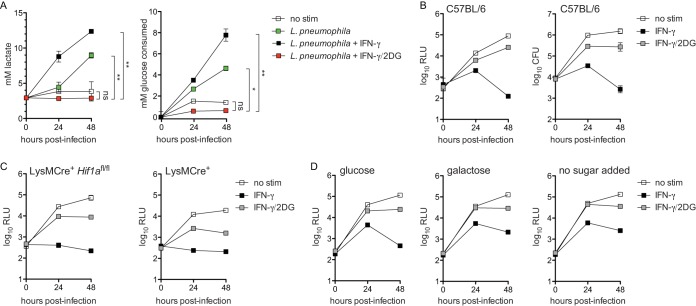
2DG rescues L. pneumophila replication in IFN-γ-stimulated macrophages. (A) Lactate secretion (left) and glucose consumption (right) measured in cell culture medium following infection of wild-type C57BL/6 BMMs with LP02 Δ*flaA* Δ*uhpC lux* L. pneumophila and stimulated with 6.0 ng/ml IFN-γ and 1.0 mM 2DG as indicated. **, *P* < 0.01; *, *P* < 0.05; ns, not significant, comparing indicated curves by 2-way ANOVA. (B) Log_10_ RLU (left) and log_10_ CFU (right) of LP02 Δ*flaA* Δ*uhpC lux* L. pneumophila from infected wild-type C57BL/6 BMMs either not stimulated (no stim), stimulated with 6.0 ng/ml IFN-γ (IFN-γ), or stimulated with 6.0 ng/ml IFN-γ plus 2.0 mM 2DG (IFN-γ/2DG). *P* < 0.001, comparing all curves to each other in each graph by 2-way ANOVA. (C) Log_10_ RLU from LP02 Δ*flaA* Δ*uhpC lux* L. pneumophila from infected LysMCre^+^ *Hif1a*^fl/fl^ and LysMCre^+^ BMMs not stimulated or stimulated with 6.0 ng/ml IFN-γ and 2.0 mM 2DG as indicated. *P* < 0.01, comparing all curves to each other in each graph by 2-way ANOVA. (D) Log_10_ RLU from LP02 Δ*flaA* Δ*uhpC lux* L. pneumophila from infected wild-type C57BL/6 BMMs stimulated with 6.0 ng/ml IFN-γ and 2.0 mM 2DG as indicated and cultured in infection medium containing 11.11 mM glucose (left) or 11.11 mM galactose in the absence of glucose (center) and in glucose-free medium with no additional source of sugar (right). *P* < 0.001, comparing all curves to each other in each graph by 2-way ANOVA. Data reflect results of individual experiments that represent at least three independent experiments. Error bars in all graphs represent standard deviation of the mean from at least three technical replicates.

We next investigated the possibility that IFN-γ may act to restrict L. pneumophila not through induction of any single antimicrobial factor but by changing the metabolic landscape of the host macrophage to be unsuitable for the metabolic needs of L. pneumophila. Specifically, we hypothesized that disruption of host macrophage glycolysis with 2-deoxyglucose (2DG) would interfere with IFN-γ-dependent restriction observed in BMMs. Macrophages infected with L. pneumophila and IFN-γ-stimulated macrophages increase rates of aerobic glycolysis, which can be measured by increased consumption of glucose and increased secretion of lactate (1, 40, 46). First, we confirmed that 2DG blocked the increased glycolysis observed in L. pneumophila-infected BMMs stimulated with IFN-γ (Fig. 2A). We next tested the implication of glycolysis inhibition in IFN-γ-stimulated BMMs in terms of L. pneumophila replication. 2DG is metabolized to 2DG-phosphate (2DGP) in mammalian cells, which is directly antimicrobial (40). However, by taking advantage of a newly identified strain of L. pneumophila resistant to the direct antimicrobial effect of 2DG(P) in BMMs (Δ*flaA* Δ*uhpC* L. pneumophila) (40), we observed that addition of 2DG to BMMs rescued L. pneumophila replication in IFN-γ-treated macrophages by 100- to 200-fold (Fig. 2B).

This result initially suggested that increased macrophage glycolysis plays a direct role in bacterial restriction. However, previous studies have demonstrated that while L. pneumophila has the capacity to metabolize glucose, it does not rely on glucose or glucose derivatives to fuel its replication in broth and is largely indifferent to perturbations in BMM glycolysis during infection (40, 47–49). To further probe the role that host macrophage glycolysis plays in terms of IFN-γ restriction of L. pneumophila, we tested other conditions under which host macrophage glycolysis is impaired. BMMs lacking hypoxia-inducible factor 1α (HIF1α) fail to upregulate glycolysis in response to inflammatory stimuli and have a defect in IFN-γ-mediated control of M. tuberculosis (22). We observed that HIF1α-deficient BMMs resembled wild-type BMMs in terms of IFN-γ-dependent restriction and 2DG rescue of L. pneumophila replication (Fig. 2C). Replacement of glucose with galactose, which inhibits increased glycolysis in IFN-γ-stimulated BMMs (22, 50), also did not alter the ability of IFN-γ to restrict or of 2DG to rescue L. pneumophila replication (Fig. 2D). Further, IFN-γ was able to mediate bacterial restriction, and 2DG was able to reverse this restriction, in BMMs cultured in glucose-free medium lacking any added sugar (Fig. 2D). Finally, we tested whether other inhibitors of glycolysis, 3-bromopyruvate (3BP) and sodium oxamate (NaO), recapitulated the effects of 2DG. Neither 3BP nor NaO reversed IFN-γ-dependent restriction of Δ*flaA* Δ*uhpC* L. pneumophila (the 2DG-resistant strain) or Δ*flaA* L. pneumophila (see Fig. S1 in the supplemental material). Together, these data indicate that glycolysis induction is not required for IFN-γ-mediated restriction of L. pneumophila replication in BMMs. This result suggests that effects of 2DG other than glycolysis inhibition are responsible for its interference with the cell-intrinsic IFN-γ-dependent immune response to L. pneumophila in BMMs.

10.1128/mBio.02629-19.1 FIG S1 3-Bromopyruvate and sodium oxamate do not rescue Δ*flaA* Δ*uhpC* L. pneumophila replication in IFN-γ-stimulated BMMs. (A) Log_10_ RLU from LP02 Δ*flaA* Δ*uhpC lux* L. pneumophila (left) and Δ*flaA lux* L. pneumophila (right) from infected wild-type C57BL/6 BMMs stimulated with 6.0 ng/ml IFN-γ ± 60.0 μM 3-bromopyruvate (3BP). (B) Log_10_ RLU from LP02 Δ*flaA* Δ*uhpC lux* L. pneumophila (left) and LP02 Δ*flaA lux* L. pneumophila (right) from infected wild-type C57BL/6 BMMs stimulated with 6.0 ng/ml IFN-γ ± 2.5 mM sodium oxamate (NaO). Data reflect results of individual experiments that represent at least three independent experiments. Error bars in all graphs represent standard deviation of the mean from at least two technical replicates. Concentrations of 3BP and NaO displayed represent the highest single point in a titration at which we observed a minimum effect on L. pneumophila replication in the absence of IFN-γ. Download FIG S1, PDF file, 0.04 MB. Copyright © 2019 Price et al. 2019 Price et al. This content is distributed under the terms of the Creative Commons Attribution 4.0 International license.

### Some, but not all, unfolded protein response stimuli reverse IFN-γ-dependent inhibition of L. pneumophila.

**FIG 3:**
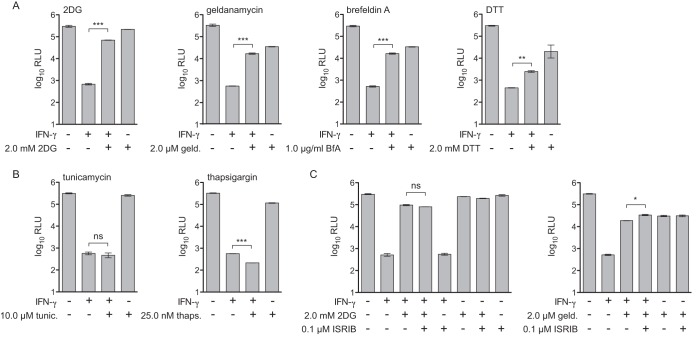
Differential effect of UPR stress stimuli on rescue of L. pneumophila replication in IFN-γ-stimulated macrophages. (A) Log_10_ RLU from LP02 Δ*flaA* Δ*uhpC lux* L. pneumophila from infected wild-type C57BL/6 BMMs stimulated for 48 h postinfection with 6.0 ng/ml IFN-γ, 2.0 mM 2DG, 2.0 μM geldanamycin (geld.), 1.0 μg/ml brefeldin A (BfA), and 2.0 mM dithiothreitol (DTT) as indicated. (B) Log_10_ RLU from LP02 Δ*flaA* Δ*uhpC lux* L. pneumophila from infected wild-type C57BL/6 BMMs stimulated for 48 h postinfection with 6.0 ng/ml IFN-γ, 10.0 μM tunicamycin (tunic.), and 25.0 nM thapsigargin (thaps.) as indicated. (C) Log_10_ RLU from LP02 Δ*flaA* Δ*uhpC lux* L. pneumophila from infected wild-type C57BL/6 BMMs stimulated for 48 h postinfection with 6.0 ng/ml IFN-γ, 2.0 mM 2DG, 2.0 μM geldanamycin, and 0.1 μM ISRIB as indicated. ***, *P* < 0.001; **, *P* < 0.01; *, *P* < 0.05; ns, not significant, comparing indicated means by unpaired *t* test. Data reflect results of individual experiments that represent at least three independent experiments. Error bars in all graphs represent standard deviation of the mean from at least two technical replicates. Concentrations of UPR stimuli displayed represent a single point in a titration at which we observed maximum effect on L. pneumophila replication in combination with IFN-γ stimulation relative to a minimum effect on L. pneumophila replication in the absence of IFN-γ.

To determine potential “off-target” effects of 2DG that could be responsible for reversal of IFN-γ-mediated restriction of L. pneumophila, we performed a transcript profiling experiment. We measured global transcript abundance in BMMs exposed to TLR2 agonist Pam3CSK4 or infected with L. pneumophila and then stimulated with IFN-γ ± 2DG. Pathway analysis of transcripts upregulated under 2DG conditions indicated induction of endoplasmic reticulum stress, also known as the unfolded protein response (UPR) (Fig. S2 and Table S1). 2DG is thought to trigger the UPR due to interference with protein glycosylation pathways in the endoplasmic reticulum (51). This led us to hypothesize that induction of the UPR perturbs IFN-γ-dependent restriction of L. pneumophila replication in BMMs. In fact, we observed that other drugs that trigger UPR stress, including geldanamycin, brefeldin A, and dithiothreitol, also rescued L. pneumophila replication in IFN-γ-stimulated BMMs by ∼10- to 50-fold (Fig. 3A). However, not all drugs that trigger the UPR rescued L. pneumophila replication in IFN-γ-treated BMMs. For example, treatment of BMMs with the potent UPR inducers tunicamycin or thapsigargin did not reverse IFN-γ-mediated restriction of L. pneumophila replication (Fig. 3B).

10.1128/mBio.02629-19.2 FIG S2 2DG triggers a transcriptional profile indicating endoplasmic reticulum stress in IFN-γ-stimulated BMMs. (A) Heat map showing fragments per kilobase million (FPKM) of differentially expressed transcripts measured by RNAseq recovered from 1.0 × 10^6^ wild-type C57BL/6 BMMs per condition stimulated for 18 hours with 50.0 ng/ml Pam3CSK4 plus 2.0 ng/ml IFN-γ ± 1.5 mM 2DG and 1.0 × 10^6^ wild-type C57BL/6 BMMs per condition infected at *T*_0_ with LP02 Δ*flaA* L. pneumophila (multiplicity of infection = 1.0) and stimulated 1 hour postinfection with 2.0 ng/ml IFN-γ ± 1.5 mM 2DG for a total of 18 hours prior to harvest. Transcript IDs, FPKM, and log_2_ fold change are shown in Table S1. Only transcripts shown to differ significantly between both Pam3CSK4/IFN-γ and L. pneumophila/IFN-γ ± 2DG conditions as calculated using TopHat/Cufflinks (64) are displayed. (B) Log_10_ *P* value of gene ontology (GO) and comprehensive resource of mammalian protein complexes (CORUM) gene sets in which transcripts significantly upregulated under +2DG conditions are enriched as determined using Metascape (66). Download FIG S2, PDF file, 0.05 MB. Copyright © 2019 Price et al. 2019 Price et al. This content is distributed under the terms of the Creative Commons Attribution 4.0 International license.

10.1128/mBio.02629-19.7 TABLE S1 Transcripts that vary significantly in BMMs exposed to Pam3CSK4 or L. pneumophila treated with IFN-γ versus IFN-γ plus 2DG. Transcripts that differ significantly (*q *< 0.05) between Pam3CSK4/IFN-γ and L. pneumophila/IFN-γ ± 2DG conditions. For each transcript, the table displays gene ID, chromosomal location, FPKM under each condition, and log_2_ fold change (IFN-γ FPKM over IFN-γ plus 2DG FPKM). Download Table S1, PDF file, 0.03 MB. Copyright © 2019 Price et al. 2019 Price et al. This content is distributed under the terms of the Creative Commons Attribution 4.0 International license.

One effect of UPR stress is arrest of protein translation via the PERK/EIF2α pathway, which can be reversed by the drug ISRIB (52). Importantly, ISRIB treatment did not interfere with 2DG- or geldanamycin-mediated rescue of L. pneumophila replication in IFN-γ-stimulated BMMs (Fig. 3C). This result indicates that reversal of IFN-γ-mediated restriction does not result from a global block in translation. We confirmed that UPR stimuli and ISRIB induced UPR-associated transcripts and inhibited ATF4-associated transcripts, respectively, via global transcript profiling (Fig. S3) (53). Taken together, these results suggest that while some UPR-triggering drugs can partially reverse IFN-γ-dependent restriction of L. pneumophila replication in BMMs, induction of the UPR is not sufficient to interfere with IFN-γ-mediated restriction. Additionally, the rescue of bacterial replication in IFN-γ-stimulated BMMs by UPR-triggering drugs does not act exclusively through general inhibition of protein translation.

10.1128/mBio.02629-19.3 FIG S3 Validation of UPR gene expression by UPR stimuli and inhibition of ATF4-dependent gene expression by ISRIB. (A) Histograms displaying transcripts per million (TPM) of *Hspa5* (heat shock protein 5, also known as BIP), *Trib3* (tribbles pseudokinase 3), *Dnajb3* (DnaJ heat shock protein family [Hsp40] member B3), *Pdia4* (protein disulfide isomerase associated 4), *Manf* (mesencephalic astrocyte-derived neurotrophic factor), and *Hyou1* (hypoxia upregulated 1) measured by RNAseq recovered from 1.0 × 10^6^ wild-type C57BL/6 BMMs per condition stimulated for 18 hours with 100 ng/ml Pam3CSK4 (Pam) alone or in combination with 6.0 ng/ml IFN-γ, IFN-γ plus 2.0 mM 2DG, IFN-γ plus 2.0 μM geldanamycin (Geld), IFN-γ plus 1.0 μg/ml brefeldin A (BfA), IFN-γ plus 2.0 mM dithiothreitol (DTT), IFN-γ plus 10.0 μM tunicamycin (Tunic), or IFN-γ plus 25.0 nM thapsigargin (Thaps). These genes are a subset associated with gene ontology term GO:0034976, response to endoplasmic reticulum stress. (B) Histograms displaying TPM of *Ddit3* (DNA-damage inducible transcript 3, also known as CHOP), *Atf3* (activating transcription factor 3), and *Asns* (asparagine synthetase) measured by RNAseq recovered from 1.0 × 10^6^ wild-type C57BL/6 BMMs per condition stimulated for 18 hours with 100 ng/ml Pam3CSK4 (Pam) alone or in combination with 6.0 ng/ml IFN-γ, IFN-γ plus 2.0 mM 2DG, or IFN-γ plus 2.0 mM 2DG plus 0.1 μM ISRIB. These genes are associated with PERK/ATF4-dependent gene expression resulting from the arrest of protein translation (53). Download FIG S3, PDF file, 0.1 MB. Copyright © 2019 Price et al. 2019 Price et al. This content is distributed under the terms of the Creative Commons Attribution 4.0 International license.

### IFN-γ fully restricts L. pneumophila in BMMs lacking IRG1 but is only partially restrictive in BMMs lacking both IRG1 and iNOS.

**FIG 4:**
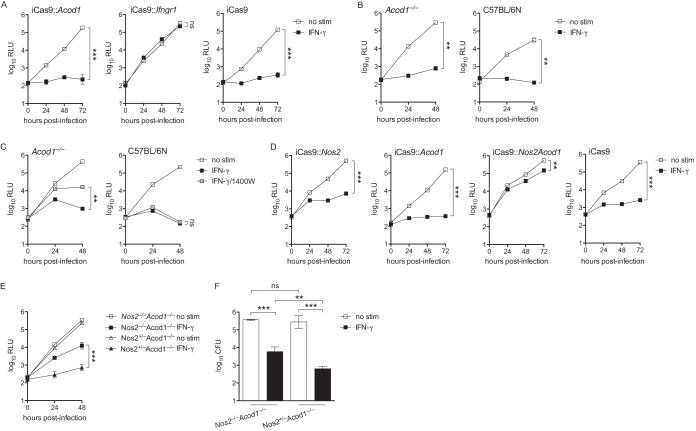
BMMs lacking IRG1 retain IFN-γ-mediated restriction of L. pneumophila while BMMs lacking both INOS and IRG1 lose the ability to fully restrict L. pneumophila. (A) Log_10_ RLU from LP02 Δ*flaA lux* L. pneumophila from infected iCas9 BMMs in which *Acod1* was targeted with two guide RNAs (iCas9::*Acod1*) or *Ifngr1* was targeted with one guide RNA (iCas9::*Ifngr1*) or that were not manipulated (iCas9) either not stimulated (no stim) or stimulated with 6.0 ng/ml IFN-γ. (B) Log_10_ RLU from LP02 Δ*flaA lux* L. pneumophila from infected primary *Acod1*^−/−^ and wild-type C57BL/6N BMMs either not stimulated or stimulated with 6.0 ng/ml IFN-γ. Wild-type C57BL/6N BMMs were included as a control for BMMs derived from *Acod1*^−/−^ mice, which were generated on the C57BL/6N background. (C) Log_10_ RLU from LP02 Δ*flaA lux* L. pneumophila from infected *Acod1*^−/−^ and wild-type C57BL/6N BMMs either not stimulated (no stim), stimulated with 6.0 ng/ml IFN-γ (IFN-γ), or stimulated with 6.0 ng/ml IFN-γ plus 100 μM 1400W (IFN-γ/1400W). (D) Log_10_ RLU from LP02 Δ*flaA lux* L. pneumophila from infected iCas9 BMMs in which *Nos2* was targeted with two guide RNAs (iCas9::*Nos2*), *Acod1* was targeted with two guide RNAs (iCas9::*Acod1*), or both *Nos2* and *Acod1* were targeted with two guide RNAs each (iCas9::*Nos2Acod1*) or that were not manipulated (iCas9) either not stimulated or stimulated with 6.0 ng/ml IFN-γ. (E) Log_10_ RLU from LP02 Δ*flaA lux* L. pneumophila from infected primary BMMs derived from *Nos2*^−/−^ *Acod1*^−/−^ and littermate *Nos2*^+/−^ *Acod1*^−/−^ mice either not stimulated or stimulated with 6.0 ng/ml IFN-γ. (A to E) ***, *P* < 0.001; **, *P* < 0.01; ns, not significant, comparing indicated curves by 2-way ANOVA. (F) Log_10_ CFU of LP02 Δ*flaA lux* L. pneumophila recovered 48 h postinfection from BMMs derived from *Nos2*^−/−^ *Acod1*^−/−^ and littermate *Nos2*^+/−^ *Acod1*^−/−^ mice either not stimulated or stimulated with 6.0 ng/ml IFN-γ. ***, *P* < 0.001; **, *P* < 0.01; ns, not significant, comparing means by unpaired *t* test. Data reflect results of individual experiments that represent at least two independent experiments. Error bars in all graphs represent standard deviation of the mean from at least three technical replicates.

Our analysis above revealed that certain drugs that trigger the UPR rescue L. pneumophila replication in IFN-γ-stimulated BMMs, while others do not. We speculated that we could use these stimuli as a filter to look for transcripts associated with a restrictive versus permissive macrophage state. Using this logic to filter results from RNA sequencing (RNAseq) analysis of BMMs stimulated with Pam3CSK4 ± IFN-γ ± UPR stimuli, we identified two genes, *Nos2* (encoding iNOS) and *Acod1* (encoding IRG1), whose transcript levels were elevated under restrictive conditions and lowered under permissive conditions (Fig. S4A). Since iNOS deficiency has no effect on IFN-γ-mediated control of L. pneumophila replication (e.g., Fig. 1B), we speculated that IRG1 may restrict L. pneumophila replication in IFN-γ-stimulated BMMs, as suggested (but not directly tested) previously (29). Using immortalized BMMs derived from C57BL/6 mice that inducibly express Cas9 (iCas9), we targeted *Acod1* and *Ifngr1* with guide RNAs to generate BMMs that lack expression of IRG1 and IFN-γ receptor, respectively (Table 1 and Fig. S5). In comparison with *Ifngr1*-targeted BMMs, which failed to restrict L. pneumophila when stimulated with IFN-γ, we observed that *Acod1*-targeted immortalized BMMs retained the ability to restrict L. pneumophila replication upon stimulation with IFN-γ (Fig. 4A). We next generated primary BMMs from *Acod1*^−/−^ mice derived on the C57BL/6NJ background (38). Similarly to immortalized BMMs, primary *Acod1*^−/−^ BMMs displayed intact IFN-γ-dependent restriction of L. pneumophila (Fig. 4B). These results suggest that IRG1 activity alone is not required for restriction of L. pneumophila in IFN-γ-stimulated macrophages.

**TABLE 1:** Guide RNAs used in this study for CRISPR

Guide RNA target	gRNA sequence	PAM^*b*^	Genotype(s) to which contributing
iCas9 BMMs^*a*^			
*Ifngr1*	GGTATTCCCAGCATACGACA	GGG	iCas9::*Ifngr1*
Irg1 (*Acod1*) exon 2	GGACAGATGGTATCATTCGG	AGG	iCas9::*Acod1*, iCas9::*Nos2Acod1*
Irg1 (*Acod1*) exon 3	GAAAAGCAGCATATGTCGGT	GGG	iCas9::*Acod1*, iCas9::*Nos2Acod1*
*Nos2* exon 2	GTCTTTCAGGTCACTTTGGT	AGG	iCas9::*Nos2*, iCas9::*Nos2Acod1*
*Nos2* intron 2-3	GTCAGTAGTGACGTCCTGAT	TGG	iCas9::*Nos2*, iCas9::*Nos2Acod1*
QKO mouse embryos			
Irg1 (*Acod1*) exon 2	TGACAGATGGTATCATTCGG	AGG	QKO/IRG1, 6KO
Irg1 (*Acod1*) exon 3	CAAAAGCAGCATATGTCGGT	GGG	QKO/IRG1, 6KO
Caspase-11 (*Casp4*) exon 5	GTATCATACTGTAGCACATC	TGG	QKO/C11, 6KO
Caspase-11 (*Casp4*) intron 4-5	ATGTTGATTTTACCGAAATG	AGG	QKO/C11, 6KO

a Requires gRNA sequence prepended with G.

b PAM, protospacer-adjacent motif.

10.1128/mBio.02629-19.4 FIG S4 *Nos2* and *Acod1* transcription segregates with conditions permissive and restrictive for L. pneumophila replication in BMMs. (A and B) Histograms displaying transcripts per million (TPM) of *Nos2*, *Acod1*, *Irgm1*, *Irgm3*, *Cybb*, and *Casp4* measured by RNAseq recovered from 1.0 × 10^6^ wild-type C57BL/6 BMMs per condition stimulated for 18 hours with 100 ng/ml Pam3CSK4 (Pam) alone or in combination with 6.0 ng/ml IFN-γ, IFN-γ plus 2.0 mM 2DG, IFN-γ plus 2.0 μM geldanamycin (geld), IFN-γ plus 1.0 μg/ml brefeldin A (BfA), IFN-γ plus 2.0 mM dithiothreitol (DTT), IFN-γ plus 10.0 μM tunicamycin (tunic), or IFN-γ plus 25.0 nM thapsigargin (thaps). Download FIG S4, PDF file, 0.1 MB. Copyright © 2019 Price et al. 2019 Price et al. This content is distributed under the terms of the Creative Commons Attribution 4.0 International license.

10.1128/mBio.02629-19.5 FIG S5 Validation of CRISPR-mediated targeting of *Acod1* in iCas9 BMMs. Western blot demonstrating loss of IRG1 protein expression in iCas9::*Acod1* BMMs when stimulated for 24 hours with either 100 ng/ml E. coli lipopolysaccharide (LPS) or 100 ng/ml Pam3CSK4 plus 10.0 ng/ml IFN-γ. IRG1 migrates at ∼53 kDa; the IRG1 antibody also stains a nonspecific band at a slightly higher molecular weight. A separate antibody was used to stain for mouse β-actin, which migrates at ∼42 kDa. Download FIG S5, PDF file, 0.3 MB. Copyright © 2019 Price et al. 2019 Price et al. This content is distributed under the terms of the Creative Commons Attribution 4.0 International license.

We next tested the hypothesis that iNOS and IRG1 activities are redundant in terms of restricting L. pneumophila in IFN-γ-stimulated BMMs. In line with this hypothesis, we observed a partial (∼10-fold) loss of restriction in *Acod1*^−/−^ BMMs treated with the iNOS inhibitor 1400W, indicating that in the absence of IRG1, iNOS function is required to mediate full restriction of L. pneumophila in IFN-γ-stimulated BMMs (Fig. 4C). Reinforcing the idea that the activities of iNOS and IRG1 are redundant in terms of the IFN-γ-coordinated response to L. pneumophila, we observed that targeting of both *Nos2* and *Acod1*, but not each factor independently, in iCas9 BMMs resulted in an ∼20-fold loss of IFN-γ-dependent restriction of L. pneumophila (comparing iCas9::*Nos2Acod1 *+ IFN-γ to iCas9::*Nos2 + *IFN-γ) (Fig. 4D). We next crossed *Nos2*^−/−^ and *Acod1*^−/−^ mice to derive littermate *Nos2*^−/−^ *Acod1*^−/−^ and *Nos2*^+/−^ *Acod1*^−/−^ mice. We observed 10- to 17-fold-greater loss of IFN-γ-dependent L. pneumophila restriction in *Nos2*^−/−^ *Acod1*^−/−^ than in *Nos2*^+/−^ *Acod1*^−/−^ BMMs (Fig. 4E and F). In sum, these results indicate that the function of either iNOS or IRG1 must be intact to mediate robust restriction of L. pneumophila replication in IFN-γ-stimulated macrophages.

### BMMs deficient in six genes are fully defective in restriction of L. pneumophila upon stimulation by IFN-γ.

**FIG 5:**
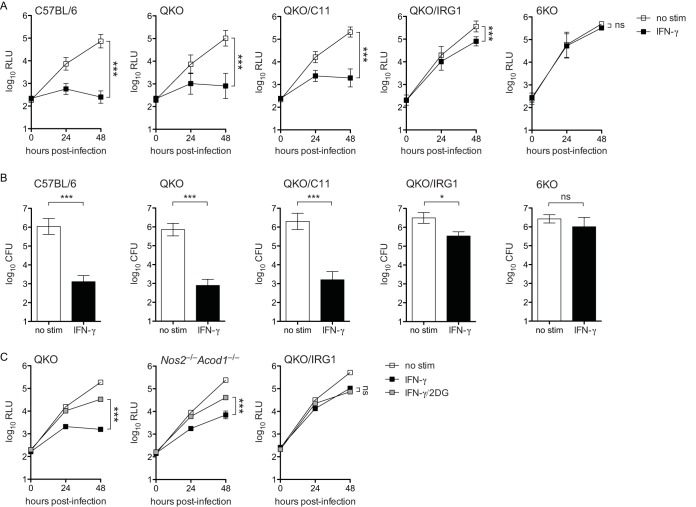
QKO BMMs that additionally lack CASP11, IRG1, or both factors display partial to complete lack of restriction of L. pneumophila replication when stimulated with IFN-γ. (A) Log_10_ RLU from LP02 Δ*flaA lux* L. pneumophila from infected wild-type C57BL/6, QKO, QKO/C11, QKO/IRG1, and 6KO BMMs either not stimulated (no stim) or stimulated with 6.0 ng/ml IFN-γ (IFN-γ). ***, *P* < 0.001; ns, not significant, comparing indicated curves by 2-way ANOVA. (B) Log_10_ CFU recovered from WT C57BL/6, QKO, QKO/C11, QKO/IRG1, and 6KO BMMs 48 h following infection with LP02 Δ*flaA lux* L. pneumophila. ***, *P* < 0.001; *, *P* < 0.05; ns, not significant, comparing means by unpaired *t* test. Data in panels A and B are pooled from multiple independent experiments using BMMs derived from two or more different mice per genotype. Error bars in panels A and B represent standard deviation of the mean as follows: C57BL/6, RLU, *n* = 5, and CFU, *n* = 5 independent experiments; QKO, RLU, *n* = 9, and CFU, *n* = 3 independent experiments; QKO/C11, RLU, *n* = 4, and CFU, *n* = 3 independent experiments; QKO/IRG1, RLU, *n* = 8, and CFU, *n* = 3 independent experiments; 6KO, RLU, *n* = 5, and CFU, *n* = 3 independent experiments. (C) Log_10_ RLU from LP02 Δ*flaA* Δ*uhpC lux* L. pneumophila from infected QKO, *Nos2*^−/−^ *Acod1*^−/−^, and QKO/IRG1 BMMs stimulated with 6.0 ng/ml IFN-γ and 2.0 mM 2DG as indicated. ***, *P* < 0.001; ns, not significant, comparing indicated curves by 2-way ANOVA. Data in panel C reflect results of individual experiments that represent at least two independent experiments.

While it appears that iNOS and IRG1 are redundant in coordinating a large proportion of L. pneumophila restriction in IFN-γ-stimulated BMMs, we observed that BMMs deficient in both iNOS and IRG1 retain partial restriction of L. pneumophila replication (Fig. 4D to F). In an effort to pinpoint the additional genetic factors that mediate IFN-γ-dependent restriction of L. pneumophila in BMMs, we made use of existing QKO mice (28). We designed an experiment to test the hypothesis that the six factors implicated across our observations (iNOS and IRG1) and the studies by Pilla et al. (28) (QKO and CASP11) and Naujoks et al. (29) (IRG1) comprise the entirety of the IFN-γ-coordinated response to L. pneumophila in macrophages. We employed CRISPR/Cas9 to target *Casp4* and *Acod1* (Table 1) in QKO mouse embryos to generate three novel mouse strains: QKO mice that also lack functional CASP11 (QKO/C11), QKO mice that also lack functional IRG1 (QKO/IRG1), and QKO mice that additionally lack both CASP11 and IRG1 (6KO). As previously reported, and in line with our observations in *Nos2* single-knockout BMMs, we observed that IFN-γ-dependent restriction of L. pneumophila in QKO BMMs was largely intact, indicating that no gene disrupted in these cells is absolutely required for restriction of L. pneumophila (Fig. 5A and B) (28). QKO/C11 BMMs did not lose IFN-γ-mediated restriction relative to QKO BMMs (Fig. 5A and B). In contrast, we observed a striking (∼100-fold) loss of restriction in QKO/IRG1 BMMs and a total loss of L. pneumophila restriction in 6KO BMMs stimulated with IFN-γ (Fig. 5A and B). We verified that QKO/IRG1 and 6KO BMMs do not display general defects in phagocytosis and endosome acidification (Fig. S6A). Additionally, we observed a significant reduction of nonpathogenic Δ*dotA* L. pneumophila (54, 55) CFU following phagocytosis by QKO/IRG1 and 6KO BMMs, indicating that early, IFN-γ-independent antimicrobial responses are intact in these macrophages (Fig. S6B).

10.1128/mBio.02629-19.6 FIG S6 Phagocytosis, endosome acidification, and capacity to reduce nonvirulent bacteria CFU in a gentamicin protection assay are intact in QKO/IRG1 and 6KO BMMs. (A) Kinetic fluorescence curves displaying fluorescent signal from pHrodo red zymosan bioparticles in wild-type C57BL/6, QKO, QKO/IRG1, and 6KO BMMs preincubated for 30 minutes with dimethyl sulfoxide (DMSO) or 1.0 μM cytochalasin D. Data reflect results of an individual experiment that is representative of two independent experiments. (B) Log_10_ CFU of LP02 Δ*dotA* L. pneumophila recovered from wild-type C57BL/6, QKO, QKO/IRG1, and 6KO BMMs following infection and 15 minutes (0 h) or 6 hours (6 h) of exposure to 100.0 μg/ml gentamicin. Data reflect results of an individual experiment that is representative of three independent experiments. ***, *P* < 0.001; **, *P* < 0.01; *, *P* < 0.05, comparing indicated means by unpaired *t* test. Download FIG S6, PDF file, 0.04 MB. Copyright © 2019 Price et al. 2019 Price et al. This content is distributed under the terms of the Creative Commons Attribution 4.0 International license.

If the ability of 2DG to rescue L. pneumophila in IFN-γ-treated BMMs acts through inhibition of iNOS and IRG1, we would expect 2DG to have no effect in BMMs lacking expression of these factors. However, we observed that 2DG retained the ability to partially rescue L. pneumophila replication (by slightly less than 10-fold) in *Nos2*^−/−^ *Acod1*^−/−^ BMMs stimulated with IFN-γ (Fig. 5C). Intriguingly, the rescue effect of 2DG was absent in QKO/IRG1 BMMs (Fig. 5C). Transcript profiling did not reveal an inhibitory effect of 2DG on expression of the other genes disrupted in the QKO/IRG1 background (Fig. S4B). This result indicates that 2DG may mediate some beneficial metabolic effect for L. pneumophila independently of regulating activity of iNOS and IRG1; however, these effects may require the other factors disrupted in the QKO background.

In sum, these results further underscore our previous observation that iNOS and IRG1 are redundant in terms of mediating a large proportion of the IFN-γ-dependent restriction of L. pneumophila in BMMs, as the addition of IRG1 deficiency to the QKO background profoundly disabled IFN-γ-mediated restriction. Further, our data reveal a partial role for CASP11 in control of L. pneumophila restriction in IFN-γ-stimulated BMMs, given the differences observed between QKO/IRG1 and 6KO BMMs. Finally, our results demonstrate that the six factors disrupted in 6KO BMMs, or a subset of those six that includes iNOS, IRG1, and CASP11, coordinate the entirety of the IFN-γ-dependent, cell-intrinsic control of L. pneumophila observed in BMMs.

## DISCUSSION

Our results support a model in which IFN-γ restricts L. pneumophila replication in mammalian macrophages through activation of multiple redundant factors, including iNOS and IRG1. To date, no study has identified any single IFN-γ-stimulated gene that fully accounts for the ability of macrophages to restrict L. pneumophila replication when stimulated with IFN-γ. Even QKO macrophages, which lack three other potentially antimicrobial factors in addition to iNOS, largely maintain the ability to restrict L. pneumophila, reinforcing the notion that redundant mechanisms contribute to IFN-γ-mediated bacterial control in macrophages.

Our recent identification of a strain of L. pneumophila resistant to the direct antimicrobial effect of 2DG when growing in BMMs (40) allowed us to test the hypothesis that global disruption of macrophage metabolism interferes with the antimicrobial effects of IFN-γ. Indeed, 2DG partially reversed the restriction of L. pneumophila replication by IFN-γ in BMMs. However, neither the glycolysis inhibition activity nor the UPR induction activity of 2DG, *per se*, appears to underlie the ability of this drug to subvert the antimicrobial effect of IFN-γ. Instead, 2DG appears to regulate the IFN-γ-dependent induction of iNOS and IRG1 via some as-yet-unidentified mechanism. In addition, there appears to be some effect of 2DG independent of iNOS and IRG1 regulation, suggesting that the drug interferes with the antimicrobial activities of IRGM1, IRGM3, and/or NOX2 in IFN-γ-stimulated macrophages. Ultimately, experimentation with 2DG and other stimuli that reversed IFN-γ-mediated restriction of L. pneumophila led us to the hypothesis that both iNOS and IRG1 are sufficient, and therefore redundant, in terms of mediating IFN-γ-coordinated immune response to L. pneumophila in macrophages.

A complex picture is emerging in terms of the role of IRG1 and the metabolite it produces, itaconate, during inflammation and infection. A direct antimicrobial role for itaconate via poisoning the bacterial glyoxylate pathway has been suggested for M. tuberculosis and L. pneumophila (29, 37). IRG1 was shown to be an essential component of the immune response to M. tuberculosis, as *Acod1*^−/−^ mice succumbed more rapidly than wild-type mice to infection (56). However, IRG1 appeared to be required for regulation of non-cell-autonomous pathological inflammation, and there was no evidence for cell-intrinsic antimicrobial effects of itaconate (56). IRG1 has also been demonstrated to be protective in a model of Zika virus infection in neurons (57). Interestingly, other studies have demonstrated anti-inflammatory effects of itaconate on myeloid cells, suggesting it may act as part of a negative feedback loop to control inflammation (39, 58). Beyond production of itaconate, the disruption of oxidative metabolic pathways caused by IRG1 activity may promote antimicrobial metabolic shifts in macrophages. Ultimately, diverse cell-intrinsic and intercellular roles for IRG1 and itaconate likely contribute to the immune response to a broad array of pathogens. Our data demonstrate that IRG1 is not essential for the cell-intrinsic immune response to L. pneumophila in macrophages treated with IFN-γ. However, our data are consistent with the observation that IRG1 activity may be sufficient to restrict L. pneumophila replication, as previously reported (29). Both NO generated by iNOS and itaconate generated by IRG1 may be directly antimicrobial to L. pneumophila in macrophages stimulated with IFN-γ. Alternately or additionally, iNOS and IRG1 may act to restrict L. pneumophila replication via coordinating global changes in macrophage metabolism that restrict access to key bacterial metabolites or otherwise render the host macrophage inhospitable for bacterial growth.

Adding IRG1 and CASP11 deficiency to the QKO background revealed further layers of redundancy in the immune response to L. pneumophila coordinated by IFN-γ. While QKO/C11 macrophages did not differ significantly from QKO macrophages in terms of IFN-γ-mediated bacterial restriction, we observed a profound loss of restriction in QKO/IRG1 macrophages, beyond what we observed in primary *Nos2*^−/−^ *Acod1*^−/−^ macrophages. This result indicates that factors other than iNOS disrupted in the QKO background may play a role in limiting L. pneumophila in IFN-γ-stimulated macrophages. For example, IRGM1-deficient macrophages displayed a partial loss of IFN-γ-dependent restriction of L. pneumophila (59). In agreement with the results of Pilla et al. (28), our data suggest that a role exists for CASP11 in the IFN-γ-mediated immune response to L. pneumophila, given the complete inability of IFN-γ to restrict L. pneumophila replication in 6KO macrophages versus QKO/IRG1 macrophages (which retain CASP11). In combination with the data showing that *Casp1/11*^−/−^ and QKO/C11 BMMs retain IFN-γ-mediated bacterial restriction, this result demonstrates that the activity of CASP11 is also redundant, at least with the activities of iNOS and IRG1. A role for CASP11 may be less apparent in our experiments using immortalized macrophages, which may be impaired in cell death pathways in addition to other major physiological differences from primary cells.

In sum, our study reveals a more comprehensive picture of the factors that are required to coordinate the IFN-γ-dependent immune response to L. pneumophila. We have not determined whether all six of the genes disrupted in 6KO BMMs cells are required to fully exert IFN-γ-dependent cell-intrinsic restriction of L. pneumophila or a subset of the six that includes iNOS, IRG1, and CASP11. Nonetheless, we are encouraged that among the numerous genes transcribed in IFN-γ-stimulated macrophages, we have narrowed the field that mediate cell-intrinsic control of L. pneumophila to six candidates. While all of the gene products disrupted in the 6KO background could function directly as antimicrobial effectors, we also note the possibility that some or all may function as upstream regulators and thus affect L. pneumophila indirectly.

IFN-γ is an essential component of the immune response to bacterial pathogens beyond L. pneumophila. Thus, the implications of this study extend beyond furthering our understanding of the immune response to L. pneumophila, an accidental pathogen of mammals that did not evolve to evade the human immune response. Our work reveals fundamental redundancy in the IFN-γ-dependent immune response to potentially pathogenic environmental microbes. Dissecting these overlapping innate immune strategies reveals the complexity and comprehensiveness of the innate immune barrier posed to novel environmental microorganisms by mammalian macrophages and IFN-γ. Further, a more detailed understanding of how IFN-γ can mediate bacterial restriction in host cells may inform studies of how “professional” pathogens, such as M. tuberculosis, S. enterica, and L. monocytogenes, have evolved to avoid or subvert these effects of IFN-γ.

## MATERIALS AND METHODS

### Ethics statement.

We conducted experiments in this study according to guidelines established by the *Guide for the Care and Use of Laboratory Animals* of the National Institutes of Health (60) under a protocol approved by the Animal Care and Use Committee at the University of California, Berkeley (AUP-2014-09-6665).

### Bone marrow-derived macrophages.

We purchased wild-type C57BL/6 (strain 000664), *Ifngr1*^−/−^ (strain 003288), *Ifnar1*^−/−^ (strain 028288), *Myd88*^−/−^ (strain 009088), *Nos2*^−/−^ (strain 002609), *Acod1*^−/−^ (strain 029340), and C57BL/6N (strain 005304) mice from Jackson Laboratory as a source of bone marrow from which to derive macrophages. *Casp1/11*^−/−^ mice were provided by A. Van der Velden and M. Starnbach (61). *Myd88*^−/−^ *Nod1*^–/–^ *Nod2*^–/–^ mice were generated at UC Berkeley as described previously (43). *Nos2*^−/−^ *Irg1*^−/−^ and *Nos2*^+/−^ *Irg1*^−/−^ mice were generated by crossing in-house at UC Berkeley. QKO mice (*Nos2*^−/−^, *Cybb*^−/−^, *Irgm1*^−/−^, and *Irgm3*^−/−^), generously provided by the lab of Christopher Sassetti at the University of Massachusetts, and mice lacking a section of chromosome 3 containing GBPs 1, 2, 3, 5, and 7 (*Gbp*^chr3−/−^), all on the C57BL/6 background, were generated as described previously (28). We generated bone marrow-derived macrophages (BMMs) in RPMI supplemented with 10% fetal bovine serum, 2.0 mM l-glutamine, and 100 μM streptomycin (all from Life Technologies) and 5% supernatant from 3T3 cells expressing macrophage colony-stimulating factor (generated in-house). Macrophages derived from LysMCre^+/+^ and LysMCre^+/+^ *Atg5*^fl/fl^ mice on the C57BL/6 background were provided by Daniel Portnoy and Jeffery Cox at UC Berkeley. Macrophages derived from LysMCre^+/+^ and LysMCre^+/+^ *Hif1a*^fl/fl^ mice on the C57BL/6 background were provided by Sarah Stanley at UC Berkeley.

### Mouse CRISPR.

We generated QKO/C11 and QKO/IRG1 mice by pronuclear injection of Cas9 mRNA and guide RNAs into fertilized embryos of QKO mice as described previously (62). Founder male mice heterozygous for mutation in either *Casp4* or *Acod1* were backcrossed once onto the QKO background, and offspring were intercrossed to generate QKO/C11, QKO/IRG1, and 6KO mice. The *Acod1* mutation was determined by amplifying a fragment of genomic DNA surrounding the cut site targeted in *Acod1* exon 2 (forward primer AACTCTGGGAATGCCAGCTC and reverse primer GGAGCCACAACAGGGATCAA, yielding an ∼440-bp PCR product) and Sanger sequencing, which revealed a three-nucleotide deletion (TTC) and a one-nucleotide insertion (A) at the cut site in mutant DNA, resulting in a frameshift mutation and premature stop codon. The *Casp4* (encoding CASP11) mutation was determined by amplifying genomic DNA surrounding the cut sites indicated by both guide RNAs (forward primer GGGGCTCTGAAAAGGTGTGA and reverse primer TCTAGACACAAAGCCCATGT, revealing an ∼520-bp band in wild-type DNA and an ∼290-bp band in mutant DNA, indicating a missing ∼230-bp fragment in mutant genomic DNA.

### iCas9 CRISPR.

We cloned template DNA for the indicated guide RNAs into a pLX-sgRNA construct additionally containing blasticidin resistance (Addgene plasmid 50662). We transfected constructs into HEK293T cells along with lentivirus packaging vector pSPAX2 (Addgene plasmid 12260) and lentivirus envelope vector VSV-G (Addgene plasmid 8454). We used the resulting virus particles to transduce immortalized wild-type C57BL/6 cells that express doxycycline-inducible SpCas9 enzyme (generated using Addgene plasmid 50661). We cultured transduced cells in 3.0 μg/ml blasticidin (InvivoGen) and 5.0 μg/ml doxycycline (Sigma) for at least 2 weeks prior to use in experiments.

### Bacterial strains, infection, and stimulation of BMMs.

LP02 is a thymidine auxotroph derived from LP01, a clinical isolate of L. pneumophila (63). Generation of Δ*flaA*, Δ*flaA* Δ*uhpC*, Δ*dotA*, and luminescent strains of L. pneumophila has been described previously (40, 41). We cultured all strains of L. pneumophila in AYE {ACES [*N*-(2-acetamido)-2-aminoethanesulfonic acid]-buffered yeast extract broth} or on ACES-buffered charcoal-yeast extract (BCYE) agar plates at 37°C. For measurement of intracellular L. pneumophila growth by luminescence or by CFU, we plated 1.0 × 10^5^ BMMs/well in opaque white TC-treated 96-well microtiter plates and infected them with L. pneumophila at a multiplicity of infection of 0.05. One hour postinfection by centrifugation at 287 × *g*, we replaced the medium of infected BMMs with medium ± stimulation at indicated concentrations. At the indicated times following infection, we measured bacterial growth by detection of luminescence at λ = 470 using a SpectraMax L luminometer (Bio-Rad) or by dilution of infected cultures on BYCE agar plates for enumeration of CFU. Pam3CSK4 and E. coli-derived lipopolysaccharide (LPS) were purchased from InvivoGen. We used 2-deoxyglucose (Abcam), brefeldin A (BD), 1400W (Cayman Chemical), 3-bromopyruvate, sodium oxamate, galactose, geldanamycin, dithiothreitol, tunicamycin, thapsigargin, and ISRIB (all from Sigma) as indicated. Recombinant mouse IFN-γ (ThermoFisher) was used at 6.0 ng/ml (60 U/ml) unless otherwise indicated. We performed lactate and glucose measurement with kits purchased from Sigma according to the manufacturer’s instructions.

### Western blotting.

Following stimulation (as indicated) for 24 h, we mixed lysates derived from 1.0 × 10^6^ BMMs per stimulation condition with SDS sample buffer (40% glycerol, 8% SDS, 2% 2-mercaptoethanol, 40 mM EDTA, 0.05% bromophenol blue, and 250 mM Tris-HCl, pH 6.8), boiled them for 5 min, and then separated them by SDS-PAGE. Rabbit anti-IRG1 antibody was from Abcam (ab222417), and mouse anti-β-actin antibody was from Santa Cruz (47778).

### Phagocytosis and endosome acidification assay.

We plated BMMs at a density of 1.0 × 10^5^ cells/well in opaque white TC-treated 96-well microtiter plates. Following pretreatment with 1.0 μM cytochalasin D (Sigma) or dimethyl sulfoxide (DMSO), we exposed BMMs to pHrodo red zymosan bioparticles (ThermoFisher) according to the manufacturer’s instructions. We measured fluorescence (530/590 excitation/emission λ) over the course of 90 min using a Synergy HT multimode microplate reader (BioTek).

### Gentamicin protection assay.

We plated BMMs at a density of 1.0 × 10^5^ cells per well in TC-treated 96-well microtiter plates and infected them with LP02 Δ*dotA* L. pneumophila at a multiplicity of infection of 10. After 1 h, we replaced the medium on infected cells and added 100 μg/ml gentamicin (Sigma). Fifteen minutes (0 h) and 6 h following gentamicin treatment, we washed BMMs three times with warm phosphate-buffered saline (PBS) and then lysed BMMs with pure water to enumerate CFU by dilution on BYCE agar plates.

### RNAseq.

We submitted RNA purified from the indicated cell culture conditions using an RNeasy kit (Qiagen) to the QB3-Berkeley Functional Genomics Laboratory, where single-read 100-bp-read-length (SR100) sequencing libraries were generated. Libraries were sequenced using either a HiSeq2500 system (Illumina) at the New York Genome Center (New York, NY) or a HiSeq4000 System (Illumina) at the Vincent J. Coates Genomics Sequencing Laboratory at UC Berkeley. We performed alignment, differential expression analysis, and gene set enrichment as described previously (64–66).

### Data availability.

We deposited the RNAseq data associated with this study in the NCBI Gene Expression Omnibus, available at https://www.ncbi.nlm.nih.gov/geo/, under accession numbers GSE135385 and GSE135386.
