# Supplementary figures and images for: IRG1 and Inducible Nitric Oxide Synthase Act Redundantly with Other Interferon-Gamma-Induced Factors To Restrict Intracellular Replication of Legionella pneumophila
Source: mBio. 2019 Nov 12;10(6):e02629-19. doi: 10.1128/mBio.02629-19 (PMC6851286; doi:10.1128/mBio.02629-19)

# FIG S1

A

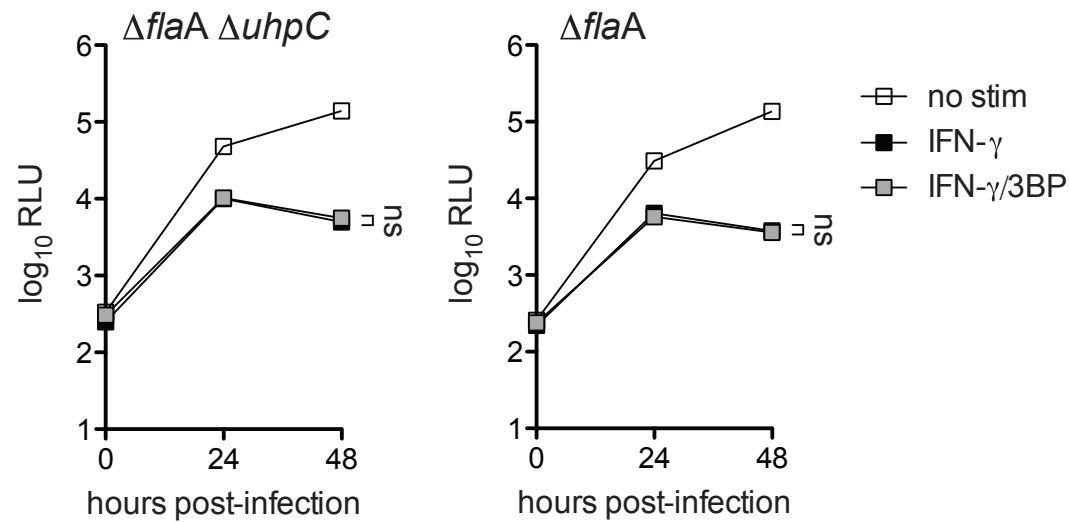

B

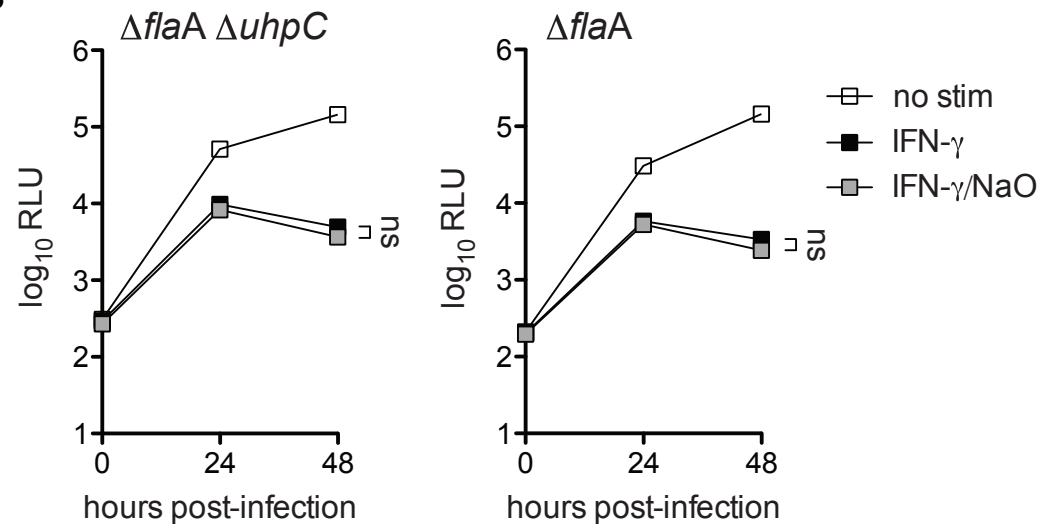

Supplement: FIG S1 [file mBio.02629-19-sf001.pdf]

# FIG S2

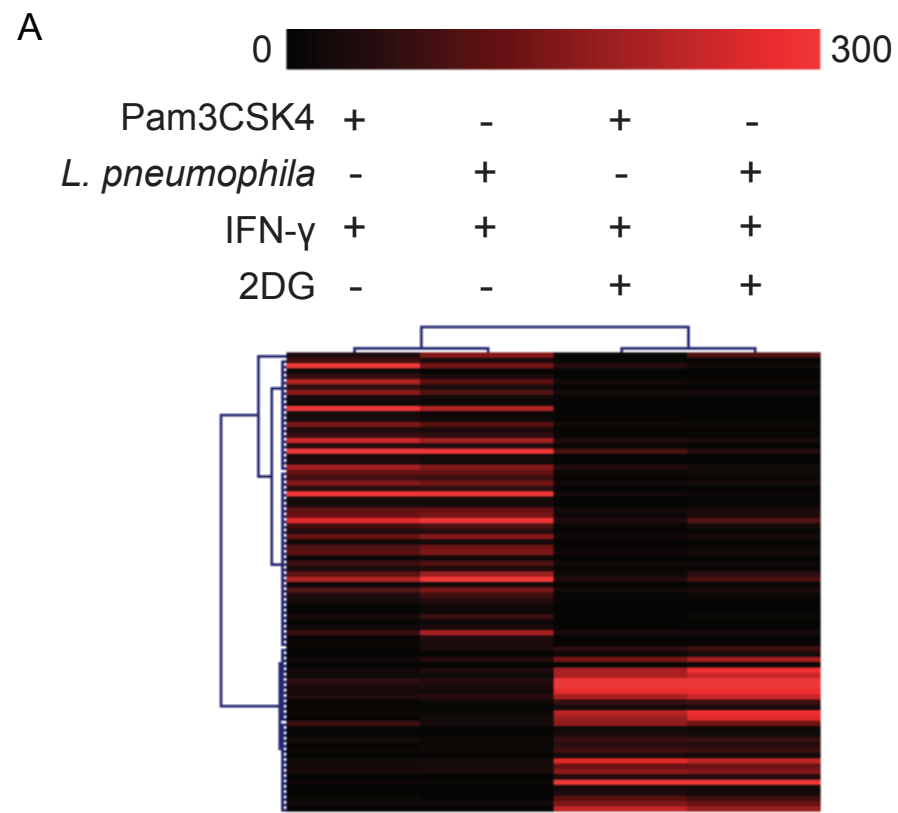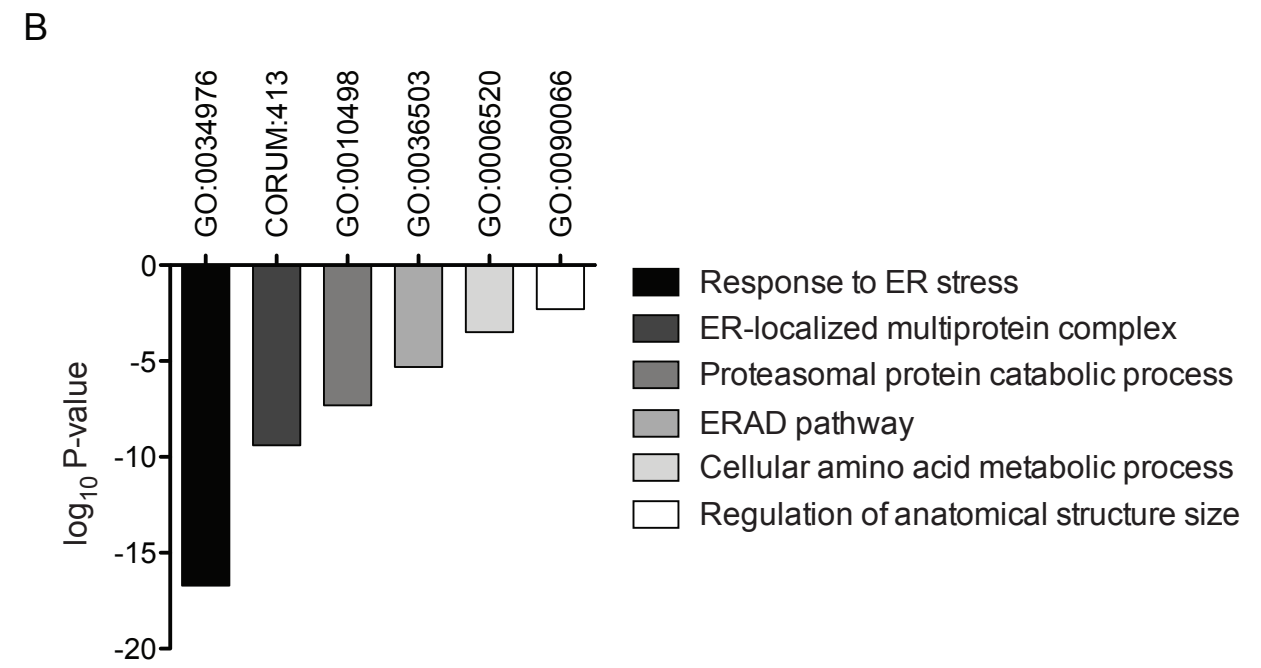

Supplement: FIG S2 [file mBio.02629-19-sf002.pdf]

FIG S3

A

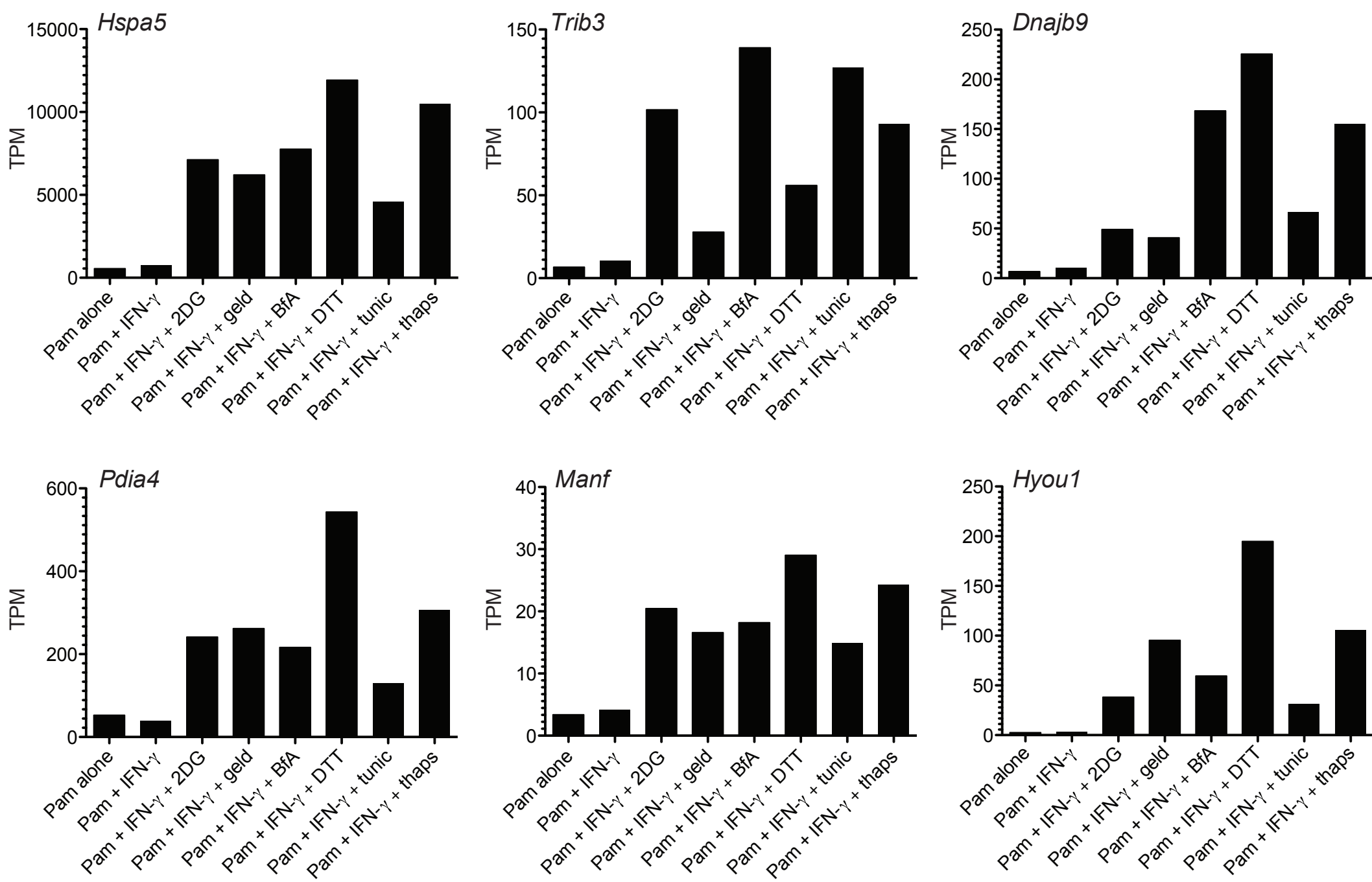

B

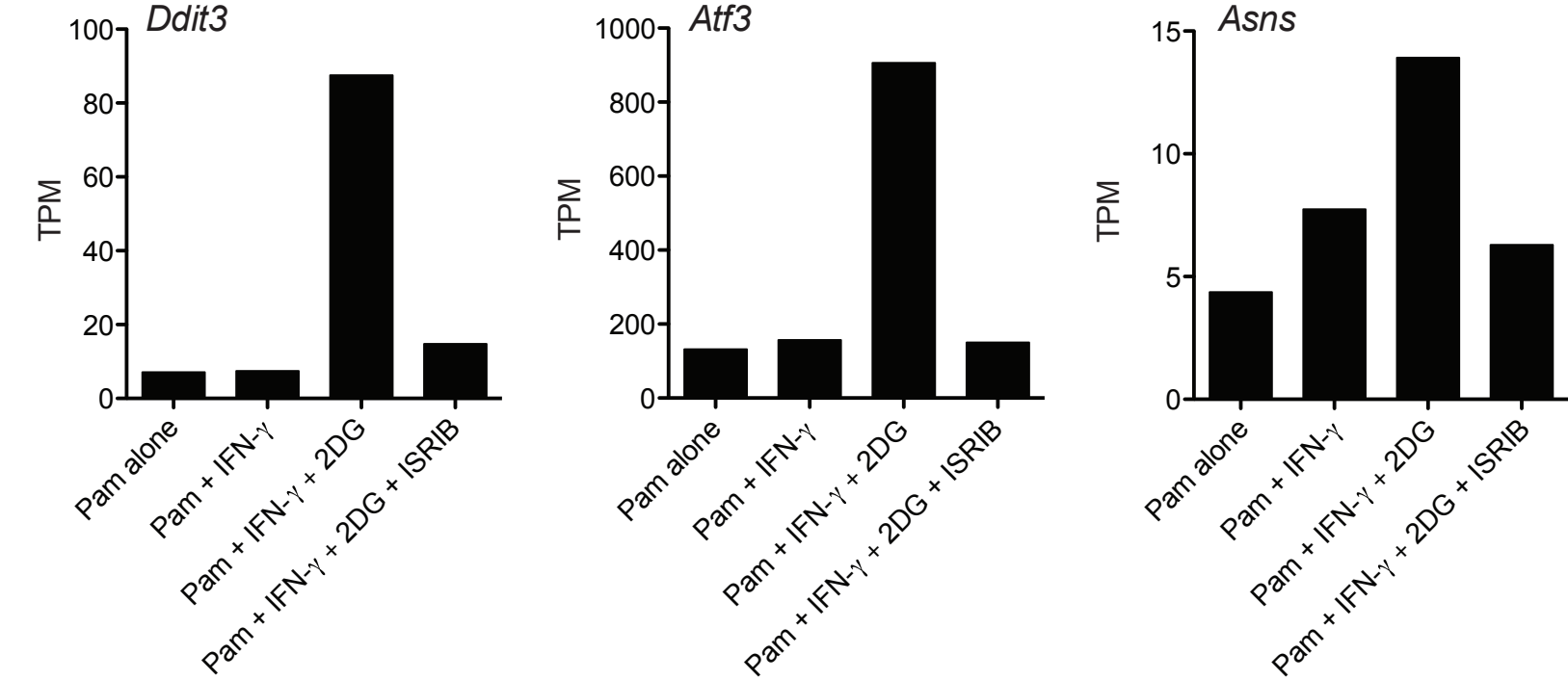

Supplement: FIG S3 [file mBio.02629-19-sf003.pdf]

FIG S4

A

*Nos2*

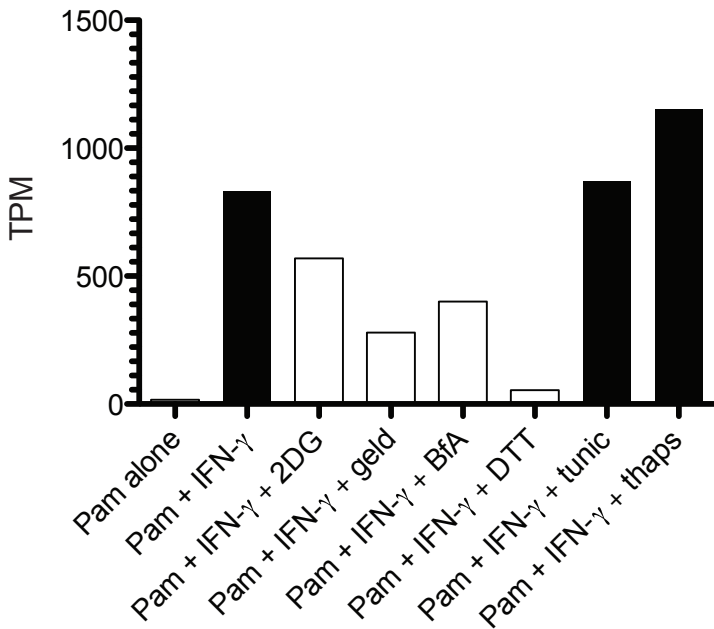

*Acod1*

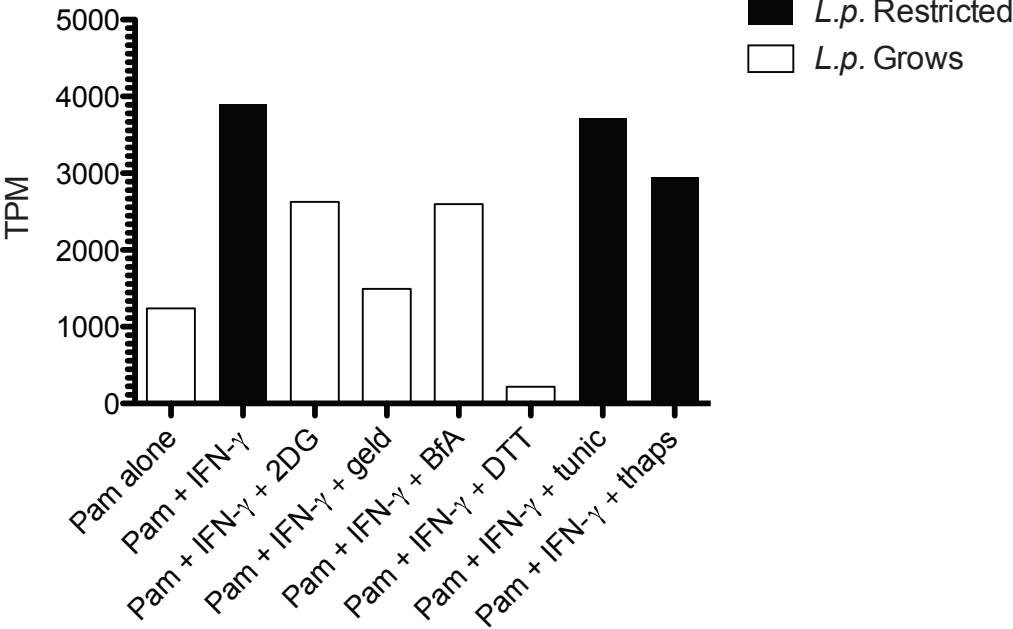

B

*Irgm1*

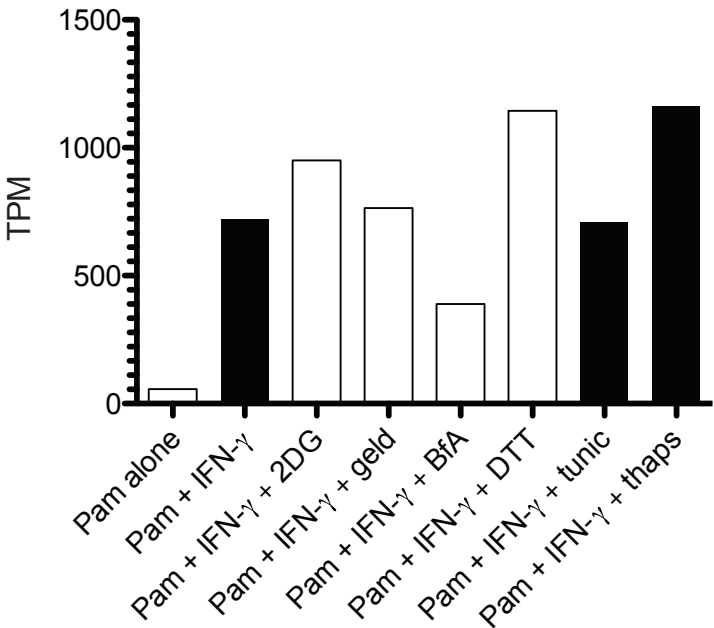

*Irgm3*

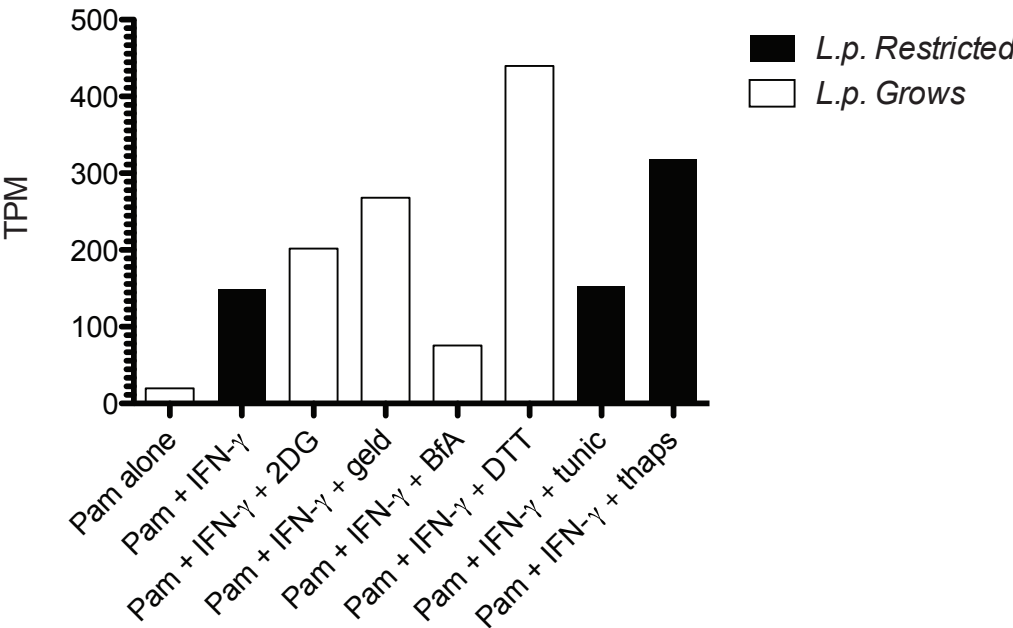

*Cybb*

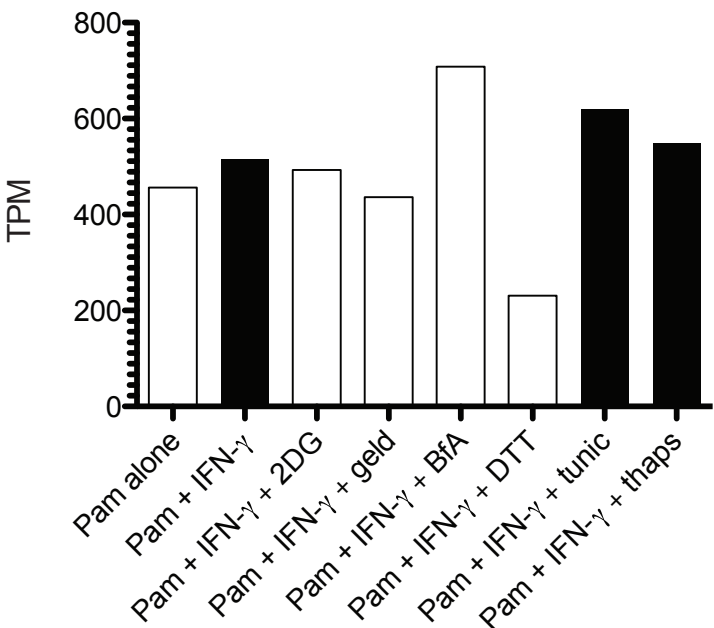

*Casp4*

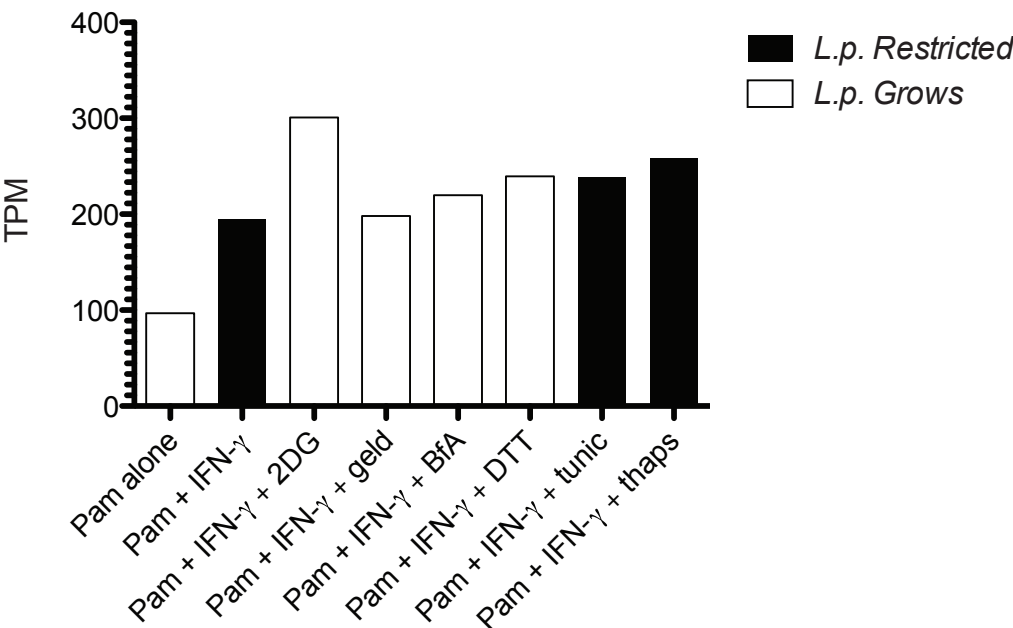

Supplement: FIG S4 [file mBio.02629-19-sf004.pdf]

FIG S5

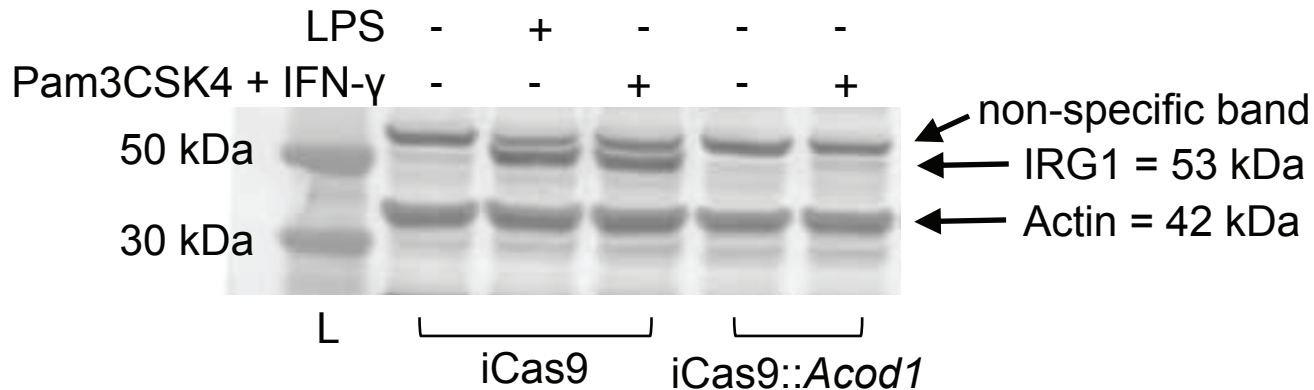

Supplement: FIG S5 [file mBio.02629-19-sf005.pdf]

FIG S6

A

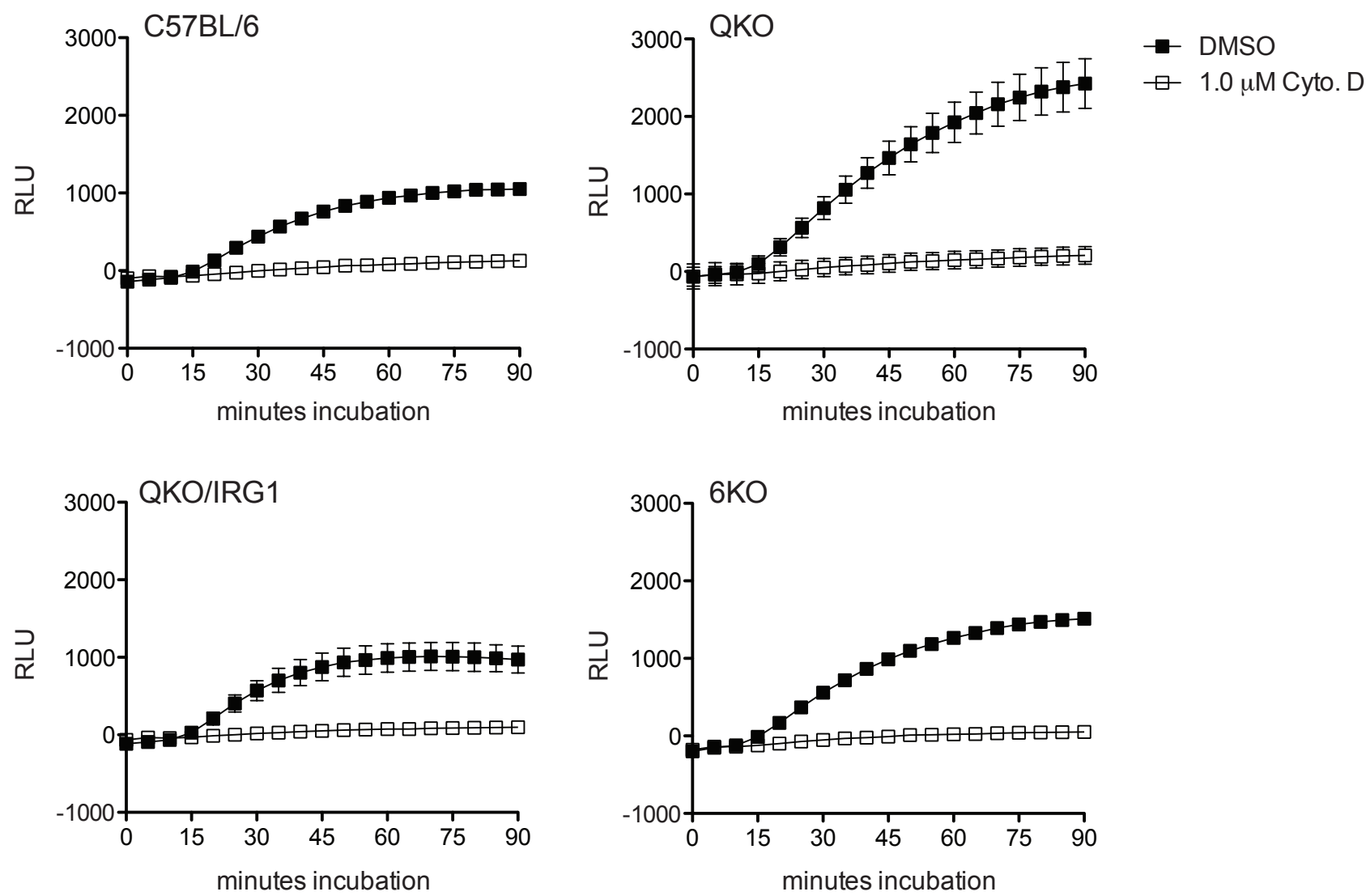

B

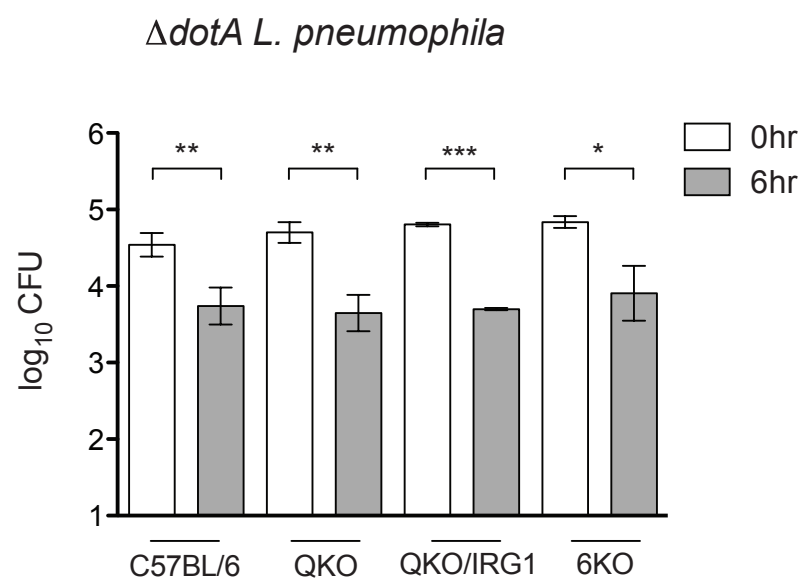

Supplement: FIG S6 [file mBio.02629-19-sf006.pdf]
