# Supplementary material for: IRG1 and Inducible Nitric Oxide Synthase Act Redundantly with Other Interferon-Gamma-Induced Factors To Restrict Intracellular Replication of Legionella pneumophila
Source: mBio. 2019 Nov 12;10(6):e02629-19. doi: 10.1128/mBio.02629-19 (PMC6851286; doi:10.1128/mBio.02629-19)
Supplement: TABLE S1 [file mBio.02629-19-st001.pdf]

Table S1. Transcripts that vary significantly in BMMs exposed to Pam3CSK4 or *L. pneumophila* treated with IFN-γ vs. IFN-γ + 2DG.

| Location | PAM + IFN-γ FPKM | *L.p.* + IFN-γ FPKM | PAM + IFN-γ + 2DG FPKM | *L.p.* + IFN-γ + 2DG FPKM | $log_2$ fold change PAM | $log_2$ fold change *L.p.* | Gene |
|---|---|---|---|---|---|---|---|
| chr3:121723536-121735052 | 8.64 | 40.80 | 0.00 | 5.34 | #DIV/0! | 2.93 | F3 |
| chr10:24914852-24927760 | 330.60 | 207.90 | 2.82 | 1.73 | 6.87 | 6.91 | Arg1 |
| chr10:99263230-99267489 | 51.60 | 57.56 | 1.67 | 2.19 | 4.95 | 4.72 | Dusp6 |
| chr11:9117979-9136170 | 378.41 | 400.15 | 22.16 | 16.15 | 4.09 | 4.63 | Upp1 |
| chr8:65618039-66473349 | 65.13 | 33.38 | 4.18 | 4.00 | 3.96 | 3.06 | March1 |
| chr12:119158487-119238276 | 19.50 | 12.96 | 1.64 | 2.44 | 3.57 | 2.41 | Itgb8 |
| chr13:60895350-60897447 | 98.59 | 132.14 | 9.22 | 13.67 | 3.42 | 3.27 | Ctla2b |
| chr2:128698860-128803160 | 22.88 | 25.86 | 2.16 | 3.90 | 3.40 | 2.73 | Mertk |
| chr13:117220572-117274415 | 81.92 | 73.16 | 7.79 | 12.61 | 3.39 | 2.54 | Emb |
| chr5:43868826-43912389 | 208.64 | 107.66 | 20.31 | 14.14 | 3.36 | 2.93 | Cd38 |
| chr14:60634728-60764556 | 26.37 | 27.59 | 2.65 | 5.08 | 3.32 | 2.44 | Spata13 |
| chr9:124102182-124109140 | 15.99 | 25.65 | 1.63 | 4.28 | 3.29 | 2.58 | Ccr2 |
| chr16:10782304-10785536 | 115.02 | 113.26 | 12.45 | 18.68 | 3.21 | 2.60 | Socs1 |
| chr7:43526933-43533175 | 30.95 | 22.50 | 3.38 | 1.66 | 3.20 | 3.76 | Cd33 |
| chr12:32173396-32208649 | 25.18 | 16.27 | 3.00 | 2.38 | 3.07 | 2.77 | Pik3cg |
| chr4:132732965-132757171 | 147.14 | 106.91 | 17.72 | 14.48 | 3.05 | 2.88 | Smpdl3b |
| chr5:123863569-123865516 | 79.28 | 95.76 | 9.69 | 20.41 | 3.03 | 2.23 | Niacr1 |
| chr10:56377299-56390419 | 76.79 | 35.79 | 9.43 | 5.28 | 3.03 | 2.76 | Gja1 |
| chr13:43785106-43803132 | 37.04 | 157.83 | 4.70 | 98.43 | 2.98 | 0.68 | Cd83 |
| chr6:29272487-29276390 | 127.10 | 143.20 | 16.23 | 38.78 | 2.97 | 1.88 | Hilpda |
| chr19:40659646-40742515 | 39.22 | 47.47 | 5.09 | 11.21 | 2.94 | 2.08 | Entpd1 |
| chr10:10335702-10472314 | 55.22 | 30.34 | 7.18 | 4.46 | 2.94 | 2.77 | Adgb |
| chr11:62248983-62266580 | 123.70 | 155.86 | 16.22 | 29.61 | 2.93 | 2.40 | Adora2b |
| chr5:43818892-43843468 | 71.44 | 57.94 | 9.40 | 9.24 | 2.93 | 2.65 | Bst1 |
| chr9:7272513-7283333 | 352.87 | 137.78 | 47.51 | 22.29 | 2.89 | 2.63 | Mmp13 |
| chr11:117965741-117969830 | 262.49 | 306.84 | 35.50 | 94.93 | 2.89 | 1.69 | Socs3 |
| chr6:123227866-123247024 | 151.03 | 133.87 | 21.24 | 26.78 | 2.83 | 2.32 | Clec4n |
| chr7:3485746-3502752 | 159.87 | 96.29 | 23.43 | 23.92 | 2.77 | 2.01 | Tarm1 |
| chr13:97241104-97253040 | 17.85 | 23.35 | 2.74 | 5.14 | 2.71 | 2.18 | Enc1 |
| chr11:83116844-83122659 | 109.82 | 148.71 | 17.36 | 26.50 | 2.66 | 2.49 | Slfn1 |
| chr17:17887823-17893952 | 569.21 | 405.70 | 90.54 | 49.52 | 2.65 | 3.03 | Fpr2 |
| chr17:17875768-17883951 | 233.86 | 190.30 | 38.79 | 25.60 | 2.59 | 2.89 | Fpr1 |
| chr12:31958478-32061279 | 31.62 | 24.11 | 5.41 | 3.49 | 2.55 | 2.79 | Prkar2b |
| chr12:85473900-85477478 | 109.40 | 181.78 | 19.23 | 57.55 | 2.51 | 1.66 | Fos |
| chr2:117279992-117342877 | 28.14 | 21.39 | 5.01 | 5.37 | 2.49 | 1.99 | Rasgrp1 |
| chr18:60393061-60443899 | 38.13 | 72.99 | 6.80 | 12.16 | 2.49 | 2.59 | EMBL-EBI AK149718.1 |
| chr15:74979722-75048837 | 203.51 | 318.72 | 37.53 | 87.21 | 2.44 | 1.87 | Ly6a |
| chr9:118606708-118901003 | 18.55 | 6.20 | 3.48 | 2.30 | 2.42 | 1.43 | Itga9 |
| chr2:164948238-164955846 | 40.93 | 31.23 | 7.80 | 7.27 | 2.39 | 2.10 | Mmp9 |
| chr16:75858793-75909273 | 96.32 | 139.27 | 18.98 | 32.44 | 2.34 | 2.10 | Samsn1 |
| chr7:38183216-38197565 | 26.84 | 48.18 | 5.34 | 5.68 | 2.33 | 3.09 | RIKEN cDNA 1600014C10 |
| chr4:132781761-132796364 | 112.02 | 119.12 | 22.44 | 31.54 | 2.32 | 1.92 | Themis2 |
| chr19:21778341-21858450 | 48.39 | 42.18 | 9.98 | 6.90 | 2.28 | 2.61 | Cemip2 |
| chr9:41012959-41157668 | 29.20 | 18.98 | 6.47 | 6.32 | 2.17 | 1.59 | Ubash3b |
| chr5:137787801-137858049 | 197.74 | 153.77 | 44.00 | 22.16 | 2.17 | 2.79 | Zcwpw1 |
| chr12:80107759-80113013 | 16.27 | 22.12 | 3.87 | 2.84 | 2.07 | 2.96 | Zfp36l1 |
| chr8:92855349-92919279 | 75.81 | 97.27 | 18.55 | 17.46 | 2.03 | 2.48 | Lpcat2 |
| chr11:48985328-48992246 | 26.32 | 69.92 | 6.72 | 13.40 | 1.97 | 2.38 | Tgtp1 |
| chr11:44454570-44470548 | 34.39 | 40.38 | 9.35 | 8.33 | 1.88 | 2.28 | Ublcp1 |
| chr7:114415253-114538029 | 9.19 | 16.69 | 2.59 | 2.62 | 1.83 | 2.67 | Pde3b |
| chr3:27317076-27339665 | 5.40 | 16.81 | 1.74 | 2.59 | 1.64 | 2.70 | Tnfsf10 |
| chr17:44096293-44105808 | 8.18 | 14.92 | 2.71 | 2.14 | 1.60 | 2.80 | Enpp4 |
| chr8:45395664-45410539 | 15.12 | 33.76 | 5.17 | 6.57 | 1.55 | 2.36 | Tlr3 |
| chr11:83175185-83190251 | 71.33 | 191.83 | 26.41 | 24.21 | 1.43 | 2.99 | Slfn4 |
| chr19:21391306-21472661 | 11.06 | 32.62 | 6.57 | 6.32 | 0.75 | 2.37 | Gda |
| chr6:7675170-7693182 | 73.21 | 24.00 | 165.25 | 132.37 | -1.17 | -2.46 | Asns |
| chr4:59805649-59904832 | 5.90 | 2.86 | 17.84 | 14.42 | -1.60 | -2.33 | Snx30 |
| chrX:36328408-36366856 | 9.53 | 4.48 | 30.18 | 22.38 | -1.66 | -2.32 | Lonrf3 |
| chr3:108424774-108445259 | 20.26 | 12.28 | 70.09 | 65.73 | -1.79 | -2.42 | Sars |
| chr3:100468062-100489192 | 3.45 | 3.65 | 14.71 | 26.76 | -2.09 | -2.87 | Fam46c |
| chr10:127514938-127522444 | 5.23 | 4.16 | 22.87 | 29.79 | -2.13 | -2.84 | Shmt2 |
| chr16:22857844-22879634 | 39.83 | 50.36 | 195.50 | 234.30 | -2.30 | -2.22 | Dnajb11 |
| chr12:116405401-116463531 | 1.97 | 2.87 | 9.82 | 14.79 | -2.32 | -2.36 | Ncapg2 |
| chr7:132557474-132576398 | 24.59 | 26.95 | 127.96 | 123.99 | -2.38 | -2.20 | Oat |
| chr11:114922780-114934386 | 9.25 | 13.23 | 53.38 | 47.90 | -2.53 | -1.86 | Cd300lb |
| chr2:121413901-121438686 | 24.63 | 23.63 | 149.03 | 124.87 | -2.60 | -2.40 | Pdia3 |
| chr12:91805907-91849157 | 26.09 | 27.07 | 159.39 | 150.35 | -2.61 | -2.47 | Sel1l |
| chr7:45522738-45526268 | 8.36 | 35.02 | 51.65 | 77.48 | -2.63 | -1.15 | Ppp1r15a |
| chr14:118937931-118981702 | 12.60 | 14.45 | 82.25 | 73.67 | -2.71 | -2.35 | Dnajc3 |
| chr13:95627176-95891922 | 3.81 | 7.23 | 27.27 | 22.09 | -2.84 | -1.61 | Iqgap2 |
| chr2:152337424-152344060 | 13.31 | 5.55 | 106.51 | 83.01 | -3.00 | -3.90 | Trib3 |
| chr18:65800577-65817657 | 16.65 | 48.02 | 140.92 | 196.46 | -3.08 | -2.03 | Sec11c |
| chr13:110395043-110400843 | 21.74 | 43.21 | 195.08 | 288.27 | -3.17 | -2.74 | Plk2 |
| chr12:17266594-17324730 | 61.82 | 47.19 | 606.19 | 664.54 | -3.29 | -3.82 | Pdia6 |
| chr14:103814624-103844524 | 2.83 | 2.98 | 27.82 | 24.59 | -3.30 | -3.04 | Ednrb |
| chr9:106887414-106891938 | 19.43 | 19.31 | 201.31 | 236.22 | -3.37 | -3.61 | Manf |
| chr6:47796140-47813512 | 31.57 | 35.74 | 330.67 | 344.11 | -3.39 | -3.27 | Pdia4 |
| chr9:44379489-44392576 | 24.95 | 23.65 | 269.93 | 281.33 | -3.44 | -3.57 | Hyou1 |
| chr16:92391952-92466146 | 5.64 | 13.03 | 63.96 | 77.68 | -3.50 | -2.58 | Rcan1 |
| chr2:84936608-84958509 | 3.33 | 2.78 | 41.74 | 39.64 | -3.65 | -3.84 | Slc43a3 |
| chr12:83987860-83993875 | 2.60 | 1.65 | 33.74 | 23.37 | -3.70 | -3.83 | Acot2 |
| chr12:44205896-44210068 | 17.95 | 25.83 | 259.96 | 223.40 | -3.86 | -3.11 | Dnajb9 |
| chr10:127290792-127311786 | 14.13 | 14.59 | 217.63 | 284.18 | -3.94 | -4.28 | Ddit3 |
| chr15:88819645-88826718 | 10.05 | 18.32 | 184.55 | 257.10 | -4.20 | -3.81 | Creld2 |
| chr16:17130137-17132383 | 15.35 | 11.96 | 331.95 | 311.42 | -4.43 | -4.70 | Sdf2l1 |
| chr8:94386499-94395358 | 6.33 | 3.36 | 225.67 | 175.88 | -5.16 | -5.71 | Herpud1 |
